# Diet-induced alteration of intestinal stem cell function underlies obesity and prediabetes in mice

**DOI:** 10.1038/s42255-021-00458-9

**Published:** 2021-09-22

**Authors:** Alexandra Aliluev, Sophie Tritschler, Michael Sterr, Lena Oppenländer, Julia Hinterdobler, Tobias Greisle, Martin Irmler, Johannes Beckers, Na Sun, Axel Walch, Kerstin Stemmer, Alida Kindt, Jan Krumsiek, Matthias H. Tschöp, Malte D. Luecken, Fabian J. Theis, Heiko Lickert, Anika Böttcher

**Affiliations:** 1 Institute of Diabetes and Regeneration Research, Helmholtz Diabetes Center, Helmholtz Center Munich, Neuherberg, Germany; 2 German Center for Diabetes Research (DZD), Neuherberg, Germany; 3 Institute of Computational Biology, Helmholtz Center Munich, Neuherberg, Germany; 4 School of Life Sciences Weihenstephan, Technical University of Munich, Freising, Germany; 5 Institute of Experimental Genetics, Helmholtz Center Munich, Neuherberg, Germany; 6 Technical University of Munich, Freising, Germany; 7 Research Unit of Analytical Pathology, Helmholtz Center Munich, Neuherberg, Germany; 8 Institute of Diabetes and Obesity, Helmholtz Diabetes Center, Helmholtz Center Munich, Neuherberg, Germany; 9 Rudolf-Buchheim-Institute of Pharmacology, Justus Liebig University, Giessen, Germany; 10 Division of Metabolic Diseases, Department of Medicine, Technical University of Munich, Munich, Germany; 11 Technical University of Munich, Munich, Germany

**Keywords:** Stem cells, Cancer, Obesity, Metabolism

## Abstract

Excess nutrient uptake and altered hormone secretion in the gut contribute to a systemic energy imbalance, which causes obesity and an increased risk of type 2 diabetes and colorectal cancer. This functional maladaptation is thought to emerge at the level of the intestinal stem cells (ISCs). However, it is not clear how an obesogenic diet affects ISC identity and fate. Here we show that an obesogenic diet induces ISC and progenitor hyperproliferation, enhances ISC differentiation and cell turnover and changes the regional identities of ISCs and enterocytes in mice. Single-cell resolution of the enteroendocrine lineage reveals an increase in progenitors and peptidergic enteroendocrine cell types and a decrease in serotonergic enteroendocrine cell types. Mechanistically, we link increased fatty acid synthesis, Ppar signaling and the Insr–Igf1r–Akt pathway to mucosal changes. This study describes molecular mechanisms of diet-induced intestinal maladaptation that promote obesity and therefore underlie the pathogenesis of the metabolic syndrome and associated complications.

## Abstract (web summary)

A combination of single-cell approaches, lineage tracing and metabolomics is used to characterize the changes to intestinal stem cell function in the small intestine that underlie intestinal maladaptation in mice fed an obesogenic diet.

## Main

Diet-induced obesity is a serious public health and economic problem. Obese people are at higher risk of developing type 2 diabetes (T2D), cardiovascular diseases and cancer, all leading causes of death worldwide (https://www.who.int). Today, bariatric surgery is the most effective treatment to achieve long-term weight loss and notably leads to diabetes remission^1^. Surgical procedures cause profound changes in secretion of gut hormones with beneficial effects on whole body metabolism, appetite and food intake^2^. These findings suggest gut hormones as candidates for new therapies against obesity and diabetes^3^.

The gut, as the body’s digestive and largest endocrine system, serves as a central regulator of energy and glucose homeostasis and quickly responds to dietary and nutritional changes^4–8^. Constant overnutrition is thought to lead to intestinal maladaptation and dysfunction and to contribute to the development of obesity and prediabetes^8^. This is evident as two hallmarks of obesity, excessive food intake and a reduced stimulation of postprandial insulin secretion by gut hormones, are linked to impaired gut function^9^. Moreover, differences in gut morphology and physiology have been observed between lean and obese individuals^8,10,11^. Tackling gut dysfunction at an early stage of disease might therefore be a promising treatment option to fight obesity and the resulting risks and complications.

Intestinal functions are carried out by specialized epithelial cells lining the gut: absorptive enterocytes, antimicrobial-peptide-secreting Paneth cells, hormone-secreting enteroendocrine cells (EECs), mucus-secreting goblet and chemosensory tuft cells. The cells of the intestinal epithelium are constantly generated from ISCs^12^. ISC identity is defined by multi-lineage potential and self-renewal capacity, but also by properties that are not hard-wired, such as the proliferative, epigenetic and metabolic state^12,13^. High cell turnover and cellular plasticity contribute to the natural adaptive capacity of the gut, but the mechanisms underlying maladaptation in response to an obesogenic diet are still unclear. Specifically, we do not know whether the hormonal imbalance and increased absorptive capacity emerge at the level of early lineage commitment from ISCs^9,14^. Moreover, the functions of enterocytes and EECs differ between gut regions^15–17^. Proximal enterocytes, for instance, are specialized to absorb iron and nutrients (carbohydrates, fat and protein), whereas distal enterocytes absorb bile acids and vitamin B12. Proximal EEC types secrete serotonin and ghrelin, whereas distally located EECs preferentially secrete Glp-1 (ref. ^17^). Gut functions are also spatially compartmentalized along the crypt–villus axis. Enterocytes shift their expression profile from an antimicrobial to a nutrient absorption to an immunomodulatory program while migrating from the bottom of the villus to its tip. The spatial–functional compartmentalization of nutrient absorption is achieved through zoned expression of nutrient transporters, with the highest expression of carbohydrate and amino acid transporters in the mid-villus region and of apolipoproteins and fatty acid transporters, such as Apoa4 and Fabp1, at the villus tip^18^. Similarly, enteroendocrine cell types switch their hormone expression pattern along the crypt–villus axis^19^. Regional identities are thought to be determined at the level of the ISCs by epigenetic mechanisms, and the crypt–villus compartmentalization is at least partly established by growth-factor gradients, but it is unknown whether and how the compartmentalization of gut functions is affected by an obesogenic diet^20,21^.

Developing pharmacological approaches to counteract obesity and diabetes require in-depth understanding of the mechanisms that underlie maladaptation and endocrine dysfunction in the gut, specifically during the transition from a healthy to an obese and to a prediabetic state. In this study we combined single-cell profiling with genetic lineage labelling and tracing of ISC fate decisions and in situ metabolomics to elucidate the cellular and molecular mechanisms that underlie intestinal maladaptation to an obesogenic western-style high-fat/high-sugar diet (HFHSD) in mice.

## Results

### HFHSD remodels the intestinal mucosa and leads to obesity

**Extended Data Fig. 1:**
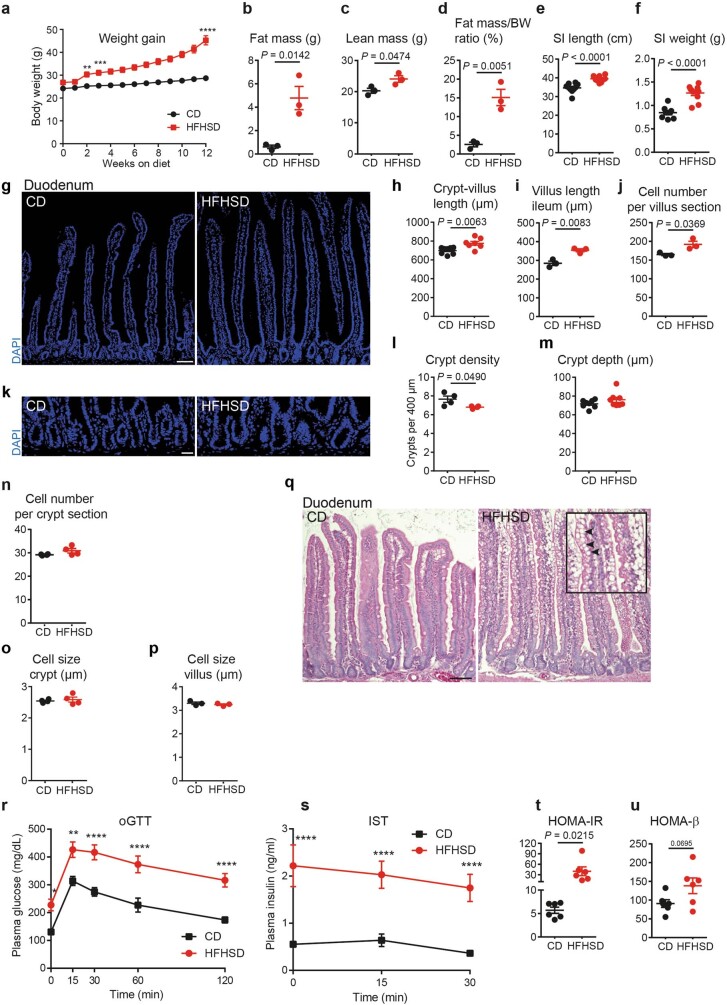
HFHSD remodels the intestinal mucosa and leads to obesity and prediabetes. **a**, Weekly assessment of body weight. *n* = 8 mice per group. Data are shown as mean ± s.e.m. of biologically independent samples. Statistical significance was determined by two-way ANOVA with Sidak’s multiple comparison test. **b**–**d**, Analysis of fat (**b**) and lean mass (**c**) by nuclear magnetic resonance (NMR) and fat mass ratio (**d**). *n* = 3 mice per group. Data are shown as mean ± s.e.m.. Statistical significance was determined by two-tailed Student’s t-test. **e**, **f**, Assessment of small intestinal length (e, *n* = 10 mice per group) and weight (f, *n* = 8 mice versus 10 mice per group). Data are shown as mean ± s.e.m. of biologically independent samples. Statistical significance was determined by two-tailed Student’s t-test. **g**–**m**, Representative LSM images of duodenal sections depicting crypt-villus units (**g**) or crypt regions (**k**). Assessment of crypt and villus morphology in duodenal and ileal sections. Duodenum: **h**, **j**, **l**, **m**. Ileum: i, *n* = 8 mice versus 7 mice (**h**), *n* = 3 mice (**i**, **j**), *n* = 4 mice (**l**) and *n* = 8 mice (**m**) per group. Data are shown as mean ± s.e.m. of biologically independent samples. Statistical significance was determined by two-tailed Student’s t-test. Scale bars, 75 µm (**g**), 25 µm (**k**). **n**–**p**, Cell number (**n**) and size (**o**) quantification in the crypts (*n* = 4 mice per group) and cell size quantification in the villi (p, *n* = 3 mice per group). Data are mean ± s.e.m. of biologically independent samples. **q**, Histological assessment of duodenal tissue sections by haematoxylin and eosin staining. Black square depicts an enlarged villus section. Black arrowheads indicate fat inclusions. Scale bar, 100 µm. *n* = 3 biologically independent CD and HFHSD samples. **r**, **s**, Assessment of glucose tolerance (**r**, *n* = 8 per group) by oral glucose tolerance test (oGTT) and insulin secretion (**s**, *n* = 4 mice versus 5 mice per group) by insulin secretion test (IST). Data are shown as mean ± s.e.m. of biologically independent samples. Statistical significance was determined by two-way ANOVA with Sidak’s multiple comparison test. **t**, **u**, Estimation of insulin resistance ((HOMA-IR) and β-cell function (HOMA-β). *n* = 6 mice per group. Data are shown as mean ± s.e.m. of biologically independent samples. Statistical significance was determined by two-tailed Student’s t-test. Source data

To study mechanisms of intestinal maladaptation to an HFHSD, we maintained male Foxa2–Venus fusion (FVF) reporter mice on a diet regimen for 12 weeks^22^. Over this time, mice on an HFHSD gained significantly more body weight (fat and lean mass) than did control diet (CD)-fed mice, and this was accompanied by an increase in length and weight of the small intestine (SI), increased villus length and decreased crypt density (Extended Data Fig. 1a–l). Crypt depth, number of cells per crypt and cell sizes in crypts and villi were not changed (Extended Data Fig. 1k,m–p). Histological assessment of the SI mucosa showed cellular fat inclusions in the villi of HFHSD-fed mice, suggesting disturbances in fatty acid metabolism in the gut epithelium (Extended Data Fig. 1q). Metabolic assessment showed that our diet-induced obese mice developed prediabetes, which was characterized by fasting hyperglycaemia, impaired glucose tolerance and pronounced hyperinsulinaemia as well as insulin resistance (Extended Data Fig. 1r–u). Thus, an HFHSD changes mucosal morphology, which is indicative of altered ISC homeostasis and lineage recruitment.

### HFHSD alters ISC lineage allocation and regional identity

**Fig. 1:**
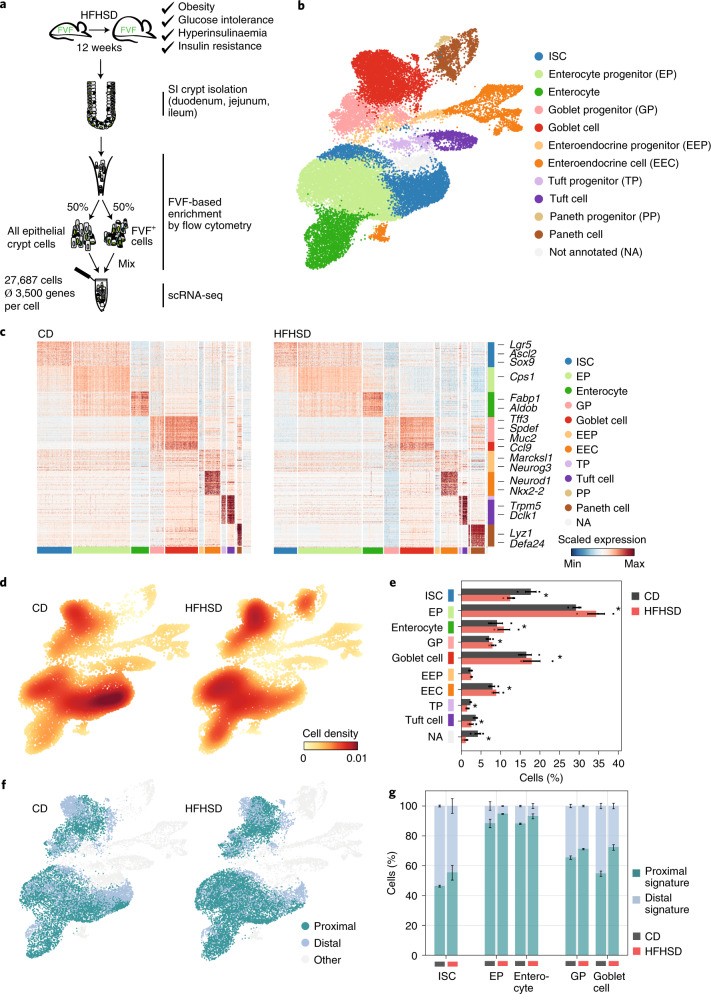
HFHSD alters lineage allocation from ISCs and shifts the regional identity of cells. **a**, Experimental design for FVF-based SI crypt-cell enrichment by flow cytometry and scRNA-seq. Single FVF^+^ and whole crypt cells from SI crypts of CD- and HFHSD-fed FVF mice were isolated in equal numbers by flow cytometry and combined for each sample. FVF-enriched single-cell samples were then transcriptionally profiled by scRNA-seq. **b**, Uniform manifold approximation and projection (UMAP) plot of 27,687 profiled single SI crypt cells. Colours highlight clustering into major intestinal cell types based on the expression of previously published marker genes. One cluster of cells could not be assigned owing to a missing marker gene signature (NA). **c**, Heatmap depicting scaled expression of cell-type-specific gene signatures in CD- and HFHSD-derived cells. Cells are represented in columns and genes are represented in rows. Colour bars indicate cell types assigned to both cells and genes. Selected known marker genes for every lineage are indicated. **d**,**e**, Cell-type composition differences in CD- and HFHSD-derived single-cell samples visualized by cell density projected onto the two-dimensional UMAP embedding (**d**) and quantified as proportions over cell types (mean ± s.e.m. of biologically independent samples, *n* = 3 mice per group, Dirichlet multinomial model) (**e**). Densities were estimated using Gaussian kernels. * Indicates a credible shift (95% highest posterior density interval of the frequency shift modelled by a Dirichlet multinomial model does not overlap 0). *n* = 3 CD mice, *n* = 3 HFHSD mice. **f**, UMAP coloured by the regional identities of cells in ISCs and goblet cell and enterocyte cell lineage. Subclusters were classified on the basis of expression of regional marker genes. **g**, Proportions of cells with proximal or distal identity in ISCs and enterocyte and goblet cell lineage (mean ± s.e.m. of biologically independent samples). *n* = 3 CD mice, *n* = 3 HFHSD mice. Source data

**Extended Data Fig. 2:**
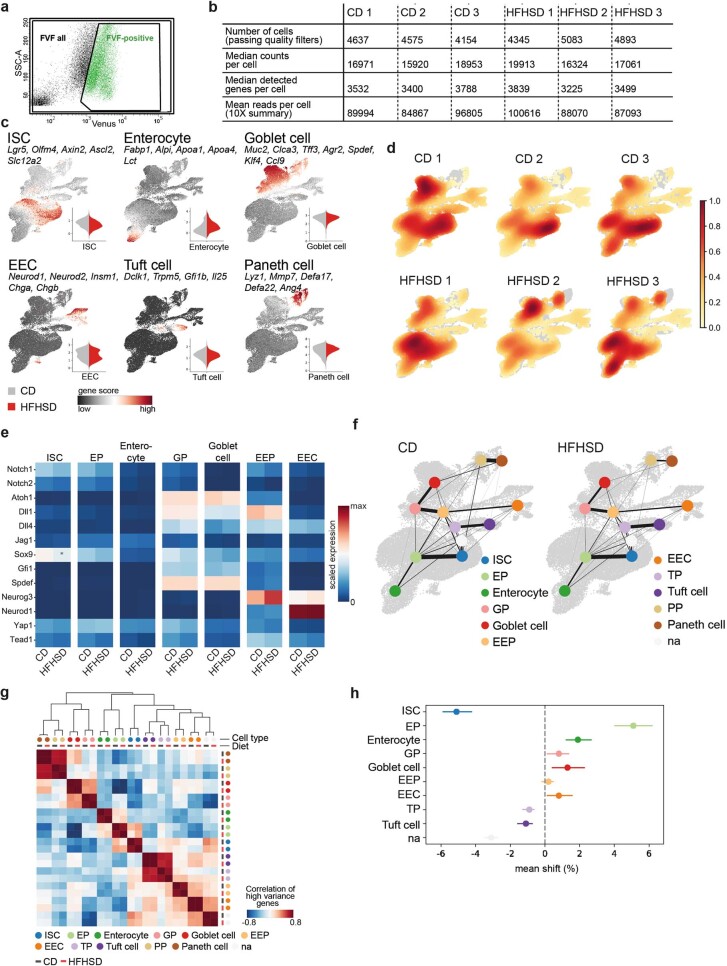
Characterization of small intestinal epithelial cell types and lineage relations in CD- and HFHSD-conditions by scRNA-seq. **a**, Representative flow cytometry plot for FVF-based enrichment of SI crypt cells for scRNA-seq. Gating strategy distinguishes FVF-positive cells from random live whole crypt cells (FVF all). **b**, Quality metrics for the scRNA-seq data. **c**, Levels of cell scores calculated based on the expression of a set of known marker genes specific for each cell type and used for cluster annotation. Violin plots show distribution of score levels assigned to the respective cell type in CD- and HFHSD-derived samples. **d**, UMAP plots of crypt cells colored by cell densities of individual CD and HFHSD mice. **e**, Mean expression levels of selected genes known to regulate lineage decisions in intestinal crypt cells of CD and HFHSD mice. *indicates a significant change in HFHSD mice (limma, FDR < 0.01, logFC>0.1). **f**, Lineage relations in CD- and HFHSD-derived SI crypt cells inferred based on a measure for cluster connectivity using PAGA. Edges are weighted by significance, node positions are cluster centres in UMAP space. **g**, Pearson correlation of mean expression profiles of high variance genes broken down by cell type and diet. Rows and columns are ordered using hierarchical clustering. **h**, Frequency shifts of each cluster from HFHSD-derived samples compared to CD-derived samples estimated using a Dirichlet-Multinomial model. Expected mean and the 95% highest posterior density interval (HPD-region) of the frequency shift for each cell type is shown. A credible shift is assumed if the HPD-region does not include 0. *n* = 3 CD mice. *n* = 3 HFHSD mice.

**Extended Data Fig. 3:**
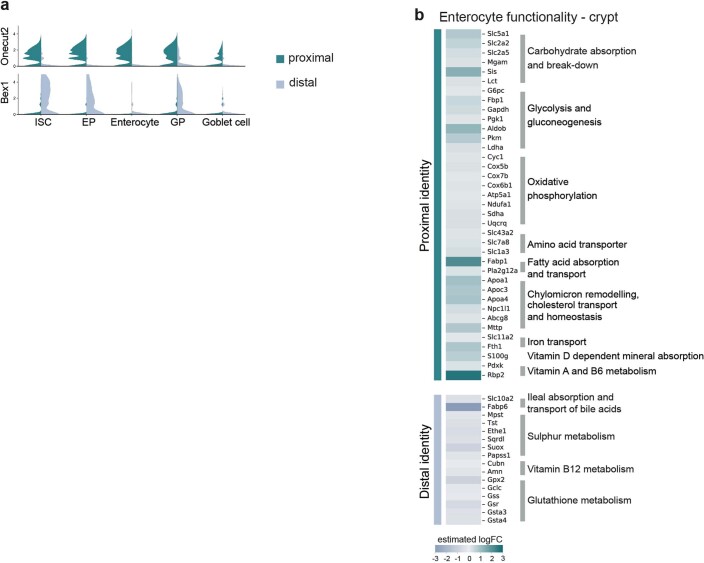
Proximal- and distal-type enterocytes exert different functions. **a**, Violin plots showing expression distribution of regional markers *Onecut2* (proximal) and *Bex1* (distal) used to classify proximal and distal cells^17^ (see Methods). **b**, Genes and their associated function characteristic for enterocytes with proximal or distal identity, respectively. Heatmap shows estimated fold change in proximal versus distal cells from CD-derived samples.

To dissect the metabolic impact of an HFHSD on ISC lineage recruitment, we employed single-cell RNA-sequencing (scRNA-seq) of crypt cells from the SI of CD- and HFHSD-fed FVF reporter mice (Fig. 1a). FVF-lineage labelling enabled us to flow sort and enrich for ISCs and EECs, cell types that together usually make up less than 6% of the intestinal epithelial cells (Fig. 1a and Extended Data Fig. 2a)^17^. This enrichment strategy not only allowed us to assess compositional changes on an HFHSD, but also to molecularly and functionally characterize rare crypt-cell types. We profiled 27,687 cells obtained from three biological replicates of CD- and HFHSD-fed mice and detected on average 3,500 genes per cell (Extended Data Fig. 2b). Unsupervised graph-based clustering and annotation based on known marker genes revealed all mature intestinal cell lineages as well as ISCs and distinct progenitor states for each intestinal lineage (Fig. 1b,c and Extended Data Fig. 2c,d). Lineage marker gene expression as well as the expression of known regulators that drive intestinal lineage decisions toward the absorptive (for example *Notch1*) and secretory lineages (*Dll1*, *Dll4* and *Atoh1*) and PAGA topology^23^ were unchanged between diet conditions (Fig. 1c, Extended Data Fig. 2c–f and Supplementary Table 1). Corresponding subtypes showed high correlation in their transcriptomes, indicating that an HFHSD did not alter lineage identities (Extended Data Fig. 2g). Instead, we observed a shift of cell densities indicating that an HFHSD impacts the composition of the mucosal epithelium (Fig. 1d,e and Extended Data Fig. 2d,h). Note that we excluded the Paneth cell lineage from the scRNA-seq analysis because of reported sampling issues, which might impact cell-type ratios^17^. To assess whether the HFHSD affects regional subtypes, we separated cell types into proximal and distal cells using recently described regional signature genes (Extended Data Fig. 3a)^17^. We found that the fractions of ISCs, enterocytes and goblet cells with proximal identity increased in mice fed an HFHSD (Fig. 1f,g and Extended Data Fig. 3b). Together, these data suggest that an HFHSD boosts formation of the absorptive and goblet cell lineage and promotes proximal cell identities, indicating that the intestine adapts cell-type composition and specific cellular functions to nutrient availability.

### Altered lineage relations in the crypt translate into the villus

**Fig. 2:**
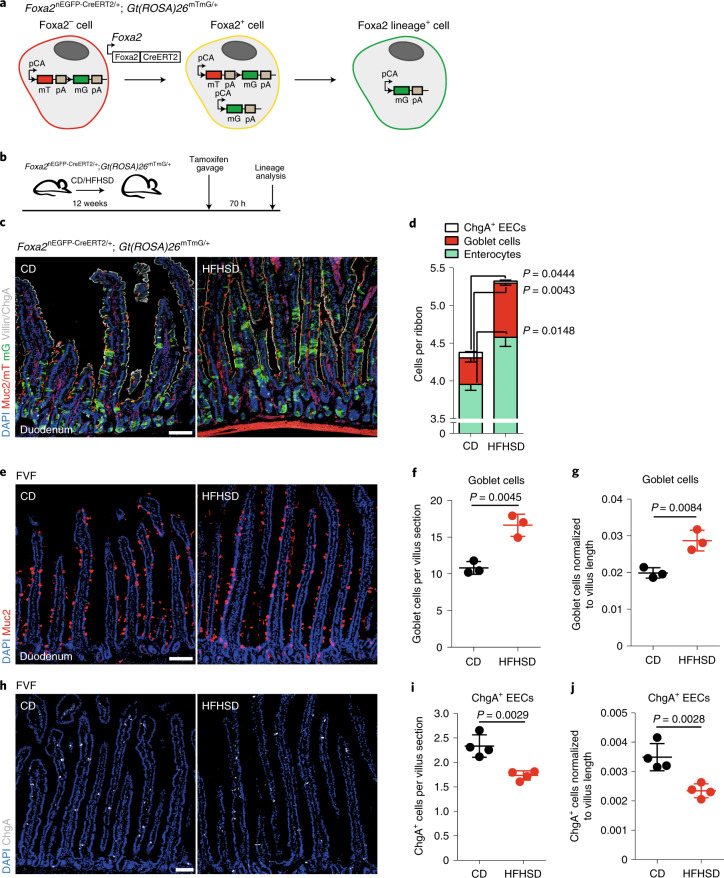
Altered lineage relations in the crypt translate into the mature villus compartment. **a**, *Foxa2*^nEGFP-CreERT2/+^*;Gt(Rosa26)*^mTmG/+^ lineage-tracing model. mT Foxa2-negative cells (red) convert into mG Foxa2-lineage-positive cells (green) upon Foxa2-promoter-driven Cre expression via an intermediate (mTmG^+^, yellow) state. pCA, chicken β-actin core promoter with a CMV enhancer. **b**, Experimental scheme of short-term lineage tracing of Foxa2-positive cells using the *Foxa2*^nEGFP-CreERT2/*+*^*;Gt(Rosa26)*^mTmG/+^ mouse model. **c**,**d**, Representative laser scanning microscopy (LSM) images of Cre-driven recombination in the duodenum of CD- and HFHSD-fed *Foxa2*^nEGFP-CreERT2/+^*;Gt(Rosa26)*^mTmG/+^ (**c**) and analysis of lineage-positive cells (**d**) 70 h after tamoxifen induction. Single converted mG^+^ cells and lineage ribbons (green) containing Muc2^+^ goblet cells (red), ChgA^+^ EECs (white) and villin^+^ enterocytes (white) are observed in crypts and villi. Scale bar, 100 µm. For Foxa2-lineage analysis, only confluent mG^+^ cell patches located in the villi were considered (*n* = 3 mice per group). Data are mean ± s.e.m. Statistical significance was determined by two-tailed Student’s *t*-test. DAPI, 4,6-diamidino-2-phenylindole. **e**–**j**, Abundances of mature intestinal cell types are altered in HFHSD-fed FVF mice. **e**,**g**, Representative LSM images (**e**) of goblet cells (Muc2^+^) and quantification thereof (**f**,**g**). Scale bar, 75 µm, *n* = 3 mice per group. **h**–**j**, Representative LSM images (**h**) of ChgA^+^ EECs and quantification thereof (**i**,**j**). Scale bar, 75 µm, *n* = 4 mice per group. Data are mean ± s.e.m. of biologically independent samples. Statistical significance was determined by two-tailed Student’s *t*-test. Source data

**Extended Data Fig. 4:**
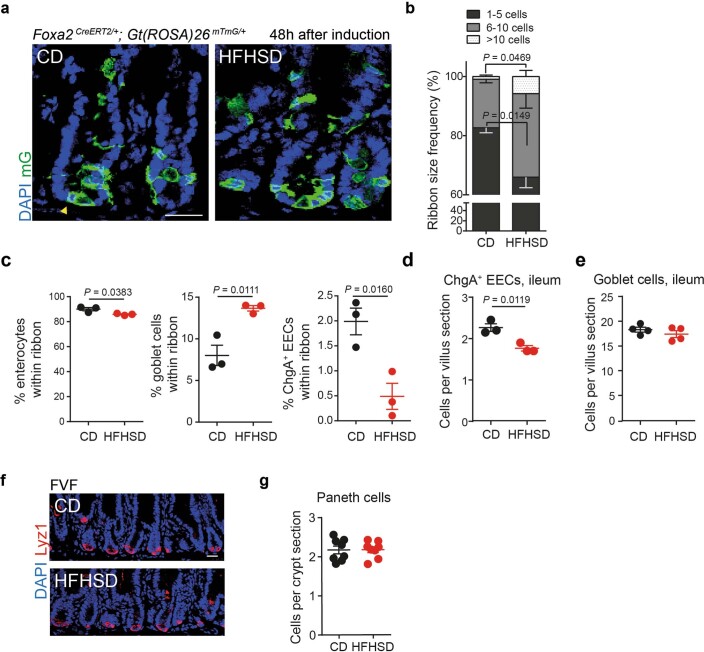
Analysis of Foxa2 lineage positive cells and frequency of mature intestinal cell types in FVF mice in CD and HFHSD conditions. **a**, Representative LSM images of tamoxifen-induced recombination in duodenal crypts of CD- and HFHSD-fed *Foxa2*^*nEGFP-CreERT2/+*^*; Gt(Rosa26)*^*mTmG/+*^ mice after 48 h. Yellow arrows indicate recombined mG^+^ slender crypt base cells (CBC) at positions typical for ISCs, adjacent to pyramid-shaped Paneth cells. Scale bar, 25 µm. *n* = 3 biologically independent CD and HFHSD samples. **b**, Frequencies of coherent Foxa2-lineage ribbons of different sizes in the villi of CD- and HFHSD-fed *Foxa2*^*nEGFP-CreERT2/+*^*; Gt(Rosa26)*^*mTmG/+*^ mice at 70 h after tamoxifen induction. *n* = 3 mice per group. Data are mean ± s.e.m. of biologically independent samples. Statistical significance was determined by two-tailed Student’s t-test. **c**, Percentage of villin^+^ enterocytes, Muc2^+^ goblet cells and ChgA^+^ EECs within Foxa2 lineage ribbons. *n* = 3 mice per group. Data are mean ± s.e.m. of biologically independent samples. Statistical significance was determined by two-tailed Student’s t-test. **d**, **e**, Quantification of ChgA^+^ EECs (**d**, *n* = 3 mice per group) and Muc2^+^ goblet cells (**e**, *n* = 4 mice per group) in ileal tissue sections of CD- and HFHSD-fed FVF mice. Data are shown as mean ± s.e.m. of biologically independent samples. Statistical significance was determined by two-tailed Student’s t-test. **f**, **g**, Representative LSM images (**f**) and quantification (**g**) of Paneth cells (Lyz1^+^) in the duodenum. Scale bar, 25 µm, n = 8 mice per group. Data are mean ± s.e.m. of biologically independent samples. Source data

**Extended Data Fig. 5:**
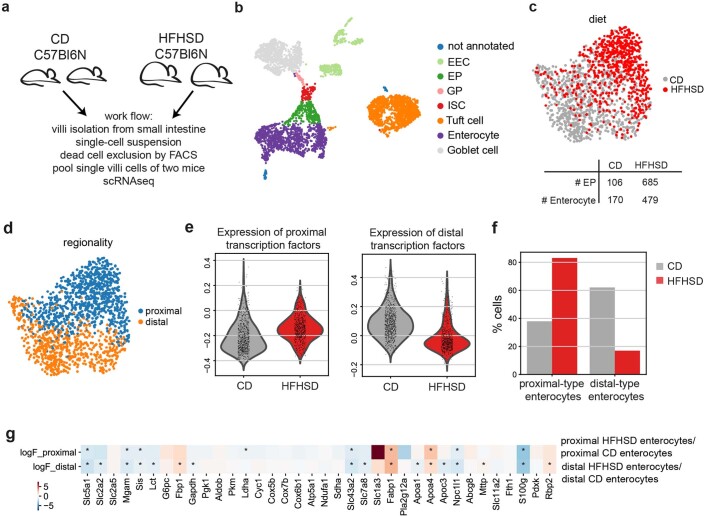
Regional identity of villi enterocytes is changed upon HFHSD. **a**, Overview of the experimental setup for scRNA-seq of villi cells. **b**, UMAP plot of 4205 profiled single SI villi cells. Colors highlight clustering into major intestinal cell types based on expression of previously published marker genes^17^. **c**, UMAP plot of enterocyte progenitors (EP) and enterocytes computed using a set of 44 transcription factors previously described to be differential between proximal and distal gut regions^17^. Colors highlight diet. **d**, UMAP plot of EP and enterocytes computed using a set of 44 transcription factors previously described to be differential between proximal and distal gut regions^17^. Colors highlight regional annotation. **e**, Violin plots showing cell scores of the proximal and distal transcription factors used in c)^17^ in CD and HFHSD-derived EPs and enterocytes. **f**, Proportions of enterocytes and EPs annotated as proximal- or distal-type enterocytes based on regional transcription factor scores in c. **g**, Heatmap showing the log2- fold change of the expression of selected functional proximal genes in HFHSD vs CD distal and proximal enterocyte cluster. *indicates a significant change. The p-values are provided in Supplementary table 1.

As our scRNA-seq experiment indicated alterations of lineage abundances in the crypt on HFHSD, we next performed short-term genetic lineage tracing of ISC fate decisions using a dual-fluorescent inducible Cre-reporter *Foxa2*^nEGFP-CreERT2/+^;*Gt(ROSA)26*^mTmG/+^ mouse (Fig. 2a)^24,25^ to determine how these changes impact the composition of the mature villus compartment. Foxa2 is expressed in quiescent and rapidly dividing ISCs (Supplementary Fig. 1). In this mouse model, *Foxa2* expression-driven CreERT2 induces a switch from membrane-Tomato (mT) to membrane-GFP (mG) in ISCs after tamoxifen administration. At 48 h after Cre-ERT2 activation by tamoxifen, we observed labelled mG-positive ISCs next to Paneth cells at the crypt bottom and labelled single cells and small cell clusters in duodenal crypts at similar frequencies in CD- and HFHSD-fed mice (Extended Data Fig. 4a). To determine the effect of an obesogenic diet on ISC lineage recruitment, we analysed the abundance of mature enterocytes, goblet cells and EECs in Cre-reporter labelled mG-positive lineage ribbons in the villi of CD- and HFHSD-fed mice 70 h after tamoxifen induction (Fig. 2b). In mice fed an HFHSD, longer ribbons (>6 cells) appeared more frequently, suggesting higher ISC or progenitor proliferation or increased cell turnover (Fig. 2c,d and Extended Data Fig. 4b). Within the lineage ribbons, total numbers of villin^+^ enterocytes and Muc2^+^ goblet cells were increased, whereas the number of ChgA^+^ EECs was reduced (Fig. 2c,d). The relative abundances of enterocytes and ChgA^+^ EECs within lineage ribbons were diminished owing to the large increase in goblet cell numbers in mice fed an HFHSD (Fig. 2c and Extended Data Fig. 4c). In accordance, goblet cell numbers in the duodenal villi were increased and numbers of ChgA^+^ EECs were reduced in the duodenum and ileum of HFHSD-fed FVF mice (Fig. 2e–j and Extended Data Fig. 4d). Note that the number of ileal goblet cells was not altered, suggesting that gut regions are differently affected by an HFHSD (Extended Data Fig. 4e). As we excluded Paneth cells from our scRNA-seq analysis, we determined the Paneth cell numbers in situ and found no difference between CD- and HFHSD-fed mice (Extended Data Fig. 4f,g). To understand whether the shift in regional identity in crypt cells translates to the villi and results in altered function, we performed scRNA-seq of villus cells from the SI (Extended Data Fig. 5a–c). We focused on enterocytes because regional and spatial compartmentalization of enterocyte function is well described and classified them into proximal and distal enterocytes using reported regional transcription factors (Extended Data Fig. 5d,e)^17,18^. Consistent with crypt scRNA-seq data, we also found an increased fraction of proximal-type villus enterocytes in mice fed an HFHSD (Extended Data Fig. 5f). Furthermore, proximal and distal-type enterocytes showed upregulation of functional proximal genes such as *Fabp1* and *Apoa4* (Extended Data Fig. 5g).

**Extended Data Fig. 6:**
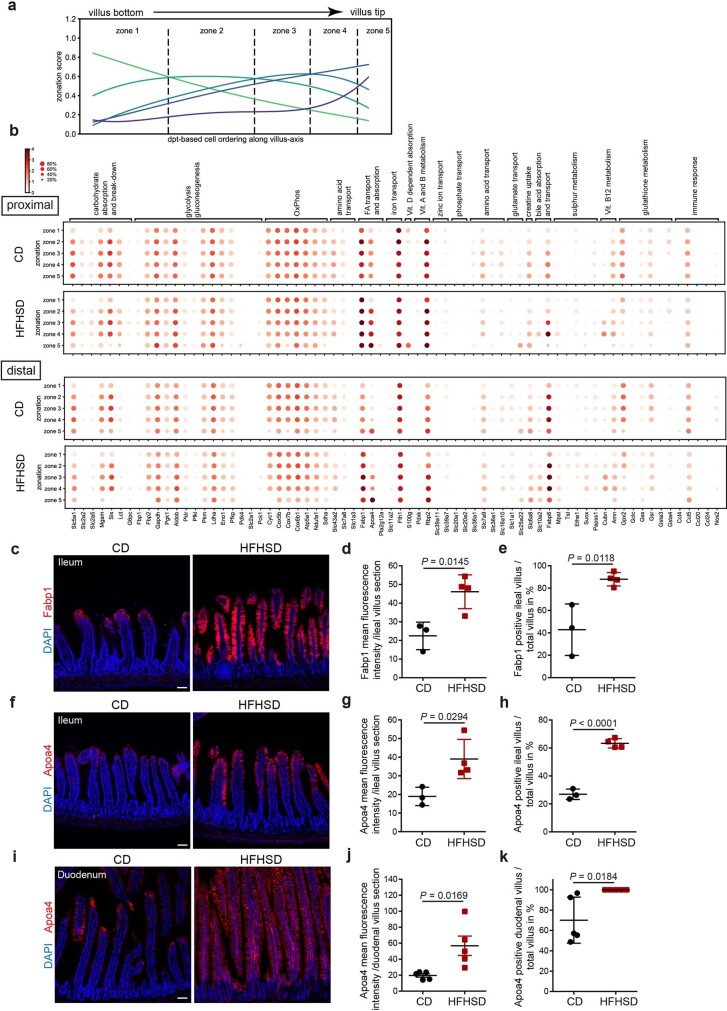
Enterocyte zonation is altered upon HFHSD. **a**, Activation of zonation scores along a pseudospatial ordering of EP and enterocytes from SI villi, which reflects the axis from villus bottom to tip. Cell scores for each zone were computed on reported markers^18^ and approximated by polynominal regression fits along the pseudospatial axis. Crossing points of the fitted lines define the partitioning into the five zones. **b**, Dot plots showing the expression of selected markers of enterocyte function in distal and proximal enterocytes. Cells are partitioned into zones along the inferred pseudospatial axis from villus bottom (zone 1) to tip (zone 5). **c**–**e**, Representative LSM images (**c**) and quantification of Fabp1 mean fluorescence intensity in ileal villi (**d**) and determination of the length of the Fabp1 positive zone (**e**). Scale bar, 50 µm, *n* = 3 CD mice and *n* = 4 HFHSD mice. Data are shown as mean ± s.d. of biologically independent samples. Statistical significance was determined by two-tailed Student’s t-test. **f**–**h**, Representative LSM images (**f**) and quantification of Apoa4 mean fluorescence intensity in ileal villi (**g**) and determination of the length of the Apoa4 positive zone (**h**). Scale bar, 50 µm, *n* = 3 CD mice and *n* = 4 HFHSD mice. Data are shown as mean ± s.d. of biologically independent samples. Statistical significance was determined by two-tailed Student’s t-test. **i**–**k**, Representative LSM images (**i**) and quantification of Apoa4 mean fluorescence intensity in duodenal villi (**j**) and determination of the length of the Apoa4 positive zone (**k**). Scale bar, 50 µm, *n* = 5 mice per group. Data are shown as mean ± s.e.m. of biologically independent samples. Statistical significance was determined by two-tailed Student’s t-test. Source data

Next, we assessed whether enterocyte zonation is affected by an HFHSD. We inferred a pseudospatial ordering of cells using previously reported zonation markers and partitioned cells into five zones along the axis from villus bottom to tip (Extended Data Fig. 6a)^18^. We found that in proximal and distal enterocytes, the spatial expression pattern of several genes associated with carbohydrate and fatty acid absorption was altered in HFHSD-fed mice (Extended Data Fig. 6b)^18^. Analysis of Fabp1 and Apoa4 immunolocalization in the ileum and duodenum confirmed the proximalization of enterocytes and altered enterocyte zonation. The expression zones of Fabp1 and Apoa4 were enlarged on an HFHSD and reached from villus tip to villus bottom (Extended Data Fig. 6c–k).

Thus, more goblet cells and proximal-type enterocytes are generated under HFHSD conditions, which leads to morphological changes of the gut mucosa and an increase in fatty acid transport and absorption. Reduced ChgA^+^ EEC numbers imply changes in specific EEC subsets.

### HFHSD changes the allocation of the enteroendocrine lineage

**Fig. 3:**
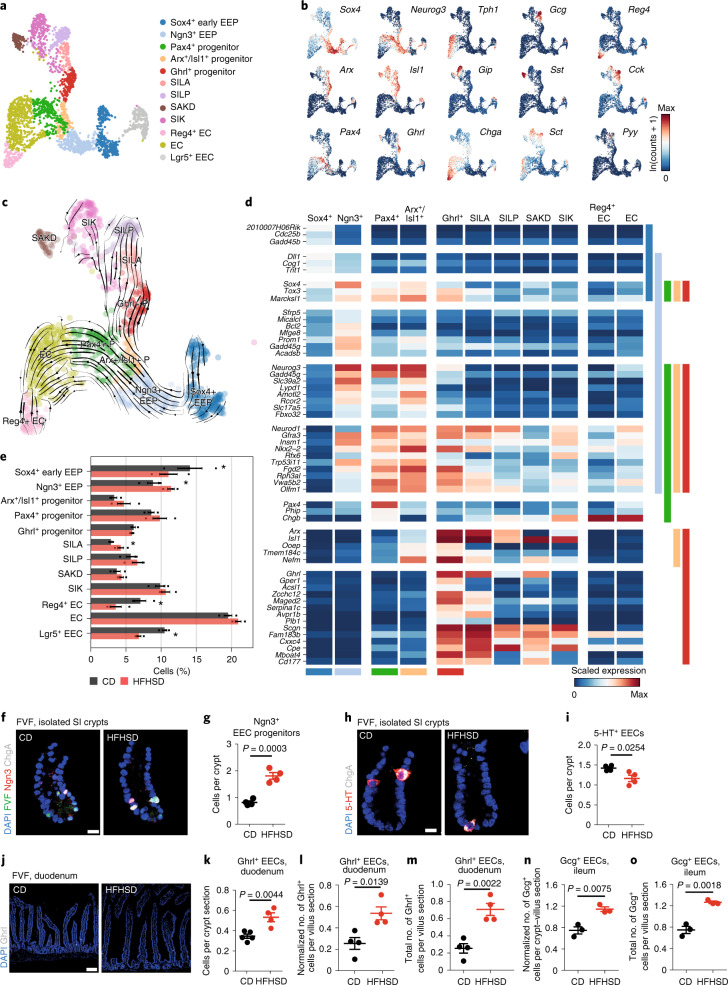
EEC lineage formation and composition in homeostasis and upon HFHSD. **a**, Colour-coded UMAP plot of 2,865 EEC lineage cells from CD- and HFHSD-derived samples. Cluster annotation was based on known marker genes and labelling of mature EEC subtypes as previously described^17^. **b**, Expression levels of selected EEC markers (hormones and transcription factors) across EEC clusters plotted in UMAP space. **c**, Streamline plot of RNA velocity projected into UMAP space showing the direction of cell differentiation along trajectories for the endocrine lineage in CD-derived samples. Arrows indicate estimated future states of cells. **d**, Heatmap showing mean expression values per cluster of genes upregulated in EEP clusters during endocrine lineage formation. Genes were selected from (pan)endocrine, lineage- and stage-specific markers (Methods). Colour bars on the side indicate expression in the progenitor populations (Fig. 3a). **e**, Cell proportions in EEC lineage subsets from CD- and HFHSD-derived samples (mean ± s.e.m. of biologically independent samples, *n* = 3 mice per group, Dirichlet multinomial model). *Indicates a credible shift (95% highest posterior density interval of the frequency shift modeled by a Dirichlet multinomial model does not overlap 0). **f**–**o**, Validation of EEC frequencies in CD- and HFHSD-fed FVF mice by immunofluorescence staining. Representative LSM images (**f**) and quantification of Ngn3^+^ cells in isolated small intestinal crypts (**g**). Scale bar, 10 µm, *n* = 4 mice per group. Representative LSM images (**h**) and quantification of 5-HT^+^ cells in isolated small intestinal crypts (**i**). Scale bar, 10 µm, *n* = 4 mice per group. Representative LSM images (**j**) and quantification of Ghrl^+^ cells in duodenal sections (**k**–**m**). Scale bar, 100 µm; crypt, *n* = 5 for CD mice and *n* = 4 HFHSD mice; villus, *n* = 4 CD and HFHSD mice. Quantification of Gcg^+^ cells in ileal sections (**n**,**o**), *n* = 3 CD and HFHSD mice. Data are shown as mean ± s.e.m. of biologically independent samples. Statistical significance was determined by two-tailed Student’s *t*-test. Source data

**Extended Data Fig. 7:**
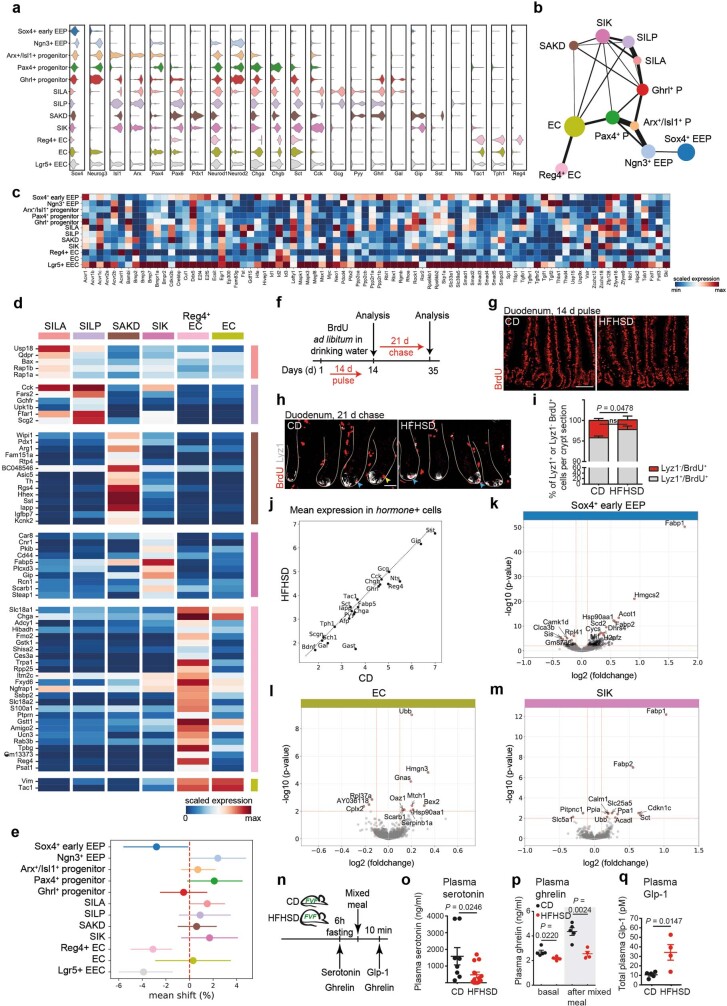
HFHSD changes transcription in EEC subsets and gut plasma hormone levels. **a**, Violin plots showing the distribution of expression of selected genes in EEC clusters. **b**, PAGA plot showing lineage relationships for the endocrine lineage in CD-derived samples. **c**, Mean expression levels per cluster of genes associated with BMP signaling. **d**, Mean expression levels per cluster of subset-specific genes in EEC clusters. **e**, Frequency shifts of each cluster from HFHSD-derived samples compared to CD-derived samples estimated using a Dirichlet-Multinomial model. Expected mean and the 95% highest posterior density interval (HPD-region) of the frequency shift for each cell type is shown. *n* = 3 mice per group. **f**–**i**, Abundance of label-retaining cells (LRCs). **f**, Schematic depicting the experimental strategy of the 5-bromo-2-deoxyuridine (BrdU) pulse-chase experiment to determine the LRC frequency. **g**, Representative LSM images of duodenal sections after 14 days of BrdU labelling. BrdU-labelled cells are stained in red. Scale bar, 50 µm. *n* = 3 biologically independent CD and HFHSD mice. **h**, Representative LSM images of duodenal crypts after a chase period of 21 days. Yellow arrowhead highlights a BrdU^+^/Lyz1^−^ LRC. Blue arrowheads indicate BrdU^+^/Lyz1^+^ cells. Scale bar, 25 µm. **i**, Proportion of Lyz1^−^ and Lyz1^+^ BrdU-labelled cells of total BrdU^+^ cells in duodenal crypts. *n* = 4 mice versus 5 mice per group. Data are presented as mean ± s.e.m. of biologically independent samples. Statistical significance was determined by two-tailed Student’s t-test. **j**, Mean hormone expression levels in EECs. Mean was calculated only from cells with high expression levels (log (count > 3). **k**–**m**, Differential gene expression in selected EEC clusters tested using limma. For each gene, the estimated logFC and its associated FDR is plotted. Red lines indicate thresholds used for significance level and gene expression change. Regulated genes are highlighted in black, of which annotated genes are the top 10 genes ranked by FDR. **n**, Experimental scheme for the assessment of selected hormone plasma levels. **o**–**q**, Plasma levels of serotonin (*n* = 4 mice per group) (**o**), ghrelin (*n* = 5 mice /4 mice per group) (**p**) and Glp-1 (*n* = 5 mice /4 mice per group) (**q**). Data represent mean ± s.e.m. of biologically independent mice. Statistical significance was determined by two-tailed Student’s t-test. Source data

Secreted gut hormones are critical regulators of food intake and systemic metabolism along the gut–brain–pancreas axis and hormonal imbalance is linked to the metabolic syndrome^26,27^. To elucidate the mechanisms underlying EEC dysfunction in response to an obesogenic diet, we first refined the clustering of our 2,865 EEC lineage cells into distinct subpopulations, which revealed five enteroendocrine progenitor (EEP) clusters characterized by expression of *Sox4*, *Ngn3*, *Arx/Isl1*, *Ghrl* and *Pax4*, respectively, and six polyhormonal EEC clusters: SILA cells (coexpress *Sct*, *Cck*, *Gcg*, *Ghrl* and *Gal*), SILP cells (coexpress *Sct*, *Cck*, *Gcg* and *Pyy*), SIK cells (coexpress *Sct*, *Cck* and *Gip*), SAKD cells (coexpress *Sct*, *Cck*, *Ghrl*, *Gip* and *Sst*), SIN cells (coexpress *Sct*, *Cck*, *Gcg* and *Nts*), enterchromaffin (EC) cells (coexpress *Sct*, *Tac1*, *Tph1*) and *Reg4*^+^ EC cells (coexpress *Sct*, *Tac1*, *Tph1*, *Ucn3* and *Reg4*) (Fig. 3a,b and Extended Data Fig. 7a,b)^17,19,28^. We also identified a population of heterogeneous EECs coexpressing endocrine and ISC markers that we termed *Lgr5*^+^ EECs and which are reminiscent of Lgr5^+^ label-retaining cells (Fig. 3a,b and Extended Data Fig. 7a)^29^. Notably, we found that *Lgr5*^+^ EECs are characterized by active Bmp signalling, which has been shown to regulate the hormonal plasticity of EECs (Extended Data Fig. 7c)^19^. To determine EEC lineage hierarchy and to understand the relationship of EEP and mature clusters, we used PAGA in combination with RNA velocity (Methods)^30^. The abstracted graph represents possible differentiation paths that cells follow and RNA velocity predicts the future state of a cell based on gene expression state (gene induction or repression), thus indicating the direction of differentiation. We observed two main differentiation trajectories from the Sox4^+^ progenitors to mature subtypes: (1) a path via *Arx*/*Isl1*^+^ and *Ghrl*^+^ progenitors to peptidergic EECs (SILA, SILP, SIK and SAKD) and (2) a route via *Pax4*^+^ progenitors to serotonergic EC and mature *Reg4*^+^ EC cells (Fig. 3c). To identify transcriptional signatures and regulators of EEC lineage allocation, we extracted genes that were either only transiently expressed in a specific EEP stage or mature cell subtype (state-specific genes) or turned on with sustained expression in subsequent states (global or lineage-specific genes) (Fig. 3d, Extended Data Fig. 7a,d and Supplementary Tables 2 and 3). Transiently expressed markers in progenitor stages (for example *Sfrp5* in *Ngn3*^+^ progenitor) are potentially important for specification. Global or lineage-specific markers (for example *Nefm* in peptidergic lineage) might regulate endocrine cell identity. Together, our data confirm EEC lineage differentiation from an early common progenitor into EC-biased (*Pax4*^+^) and non-EC-biased (*Arx*/*Isl1*^+^) progenitors and their respective molecular programs^17,28,31^. We then compared EEC lineage allocation in CD- and HFHSD-fed animals. We found that an HFHSD reduces the number of *Sox4*^+^ early EEPs, increases the fraction of *Ngn3*^+^ EEPs, reduces the number of Lyz1^−^BrdU^+^ cells that correspond to label-retaining *Lgr5*^+^ EECs and affects mature SILA and *Reg4*^+^ EC cells, which are most abundant in the duodenum (Fig. 3e–o and Extended Data Fig. 7e–i)^17^. Serotonin (5-HT)^+^*Reg4*^+^ EC numbers were decreased in HFHSD crypts, whereas Ghrl^+^ SILA cells were more abundant in the duodenal crypts and villi (Fig. 3h–m). Numbers of ileal located SILA/SILP cells expressing *Gcg*, which encodes the incretin Glp-1, were also increased with an HFHSD (Fig. 3n,o). We next assessed whether an HFHSD induces transcriptome changes that might affect EEC lineage allocation or function. Single-cell messenger RNA expression levels of hormones did not differ between CD and HFHSD EEC subsets (Extended Data Fig. 7j). However, genes associated with metabolism (for example *Slc5a1*, *Acadl* in SIK cells), vesicular trafficking machinery and the secretome (for example *Cplx2* in EC and *Sct* in SIK cells) as well as signalling (transduction) (for example *ID1/ID3* in *Sox4*^+^ progenitors, *Gnas* in EC cells) and transcription factors (for example *Cdx2* in *Sox4*^+^ progenitor, *Hmgn3* in EC cells) were differentially expressed between CD and HFHSD conditions (Extended Data Fig. 7k–m and Supplementary Table 4).

Next, we examined EEC functionality by assessing hormone secretion (Extended Data Fig. 7n). Intestinal EC cells produce over 90% of the body’s serotonin^32^. We found that basal plasma levels of serotonin were lower in mice fed an HFHSD, which correlates with reduced numbers of 5-HT^+^ EECs (*Reg4*^+^ EC cells) (Extended Data Fig. 7o). By contrast, despite a higher number of Ghrl^+^ SILA EECs, plasma levels of ghrelin were reduced in HFHSD-fed mice (Extended Data Fig. 7p), suggesting that Ghrl^+^ EECs are functionally impaired upon HFHSD. Glp-1 plasma levels were also increased, which corresponds with increased numbers of ileal Gcg^+^ cells (Extended Data Fig. 7q). Taken together, these results show that an HFHSD impacts EEPs, alters the number of specific mature EEC subtypes and circulating gut hormone levels and affects expression of genes that are potentially important for endocrine cell differentiation and/or function.

### HFHSD induces hyperproliferation of ISCs and progenitors

**Fig. 4:**
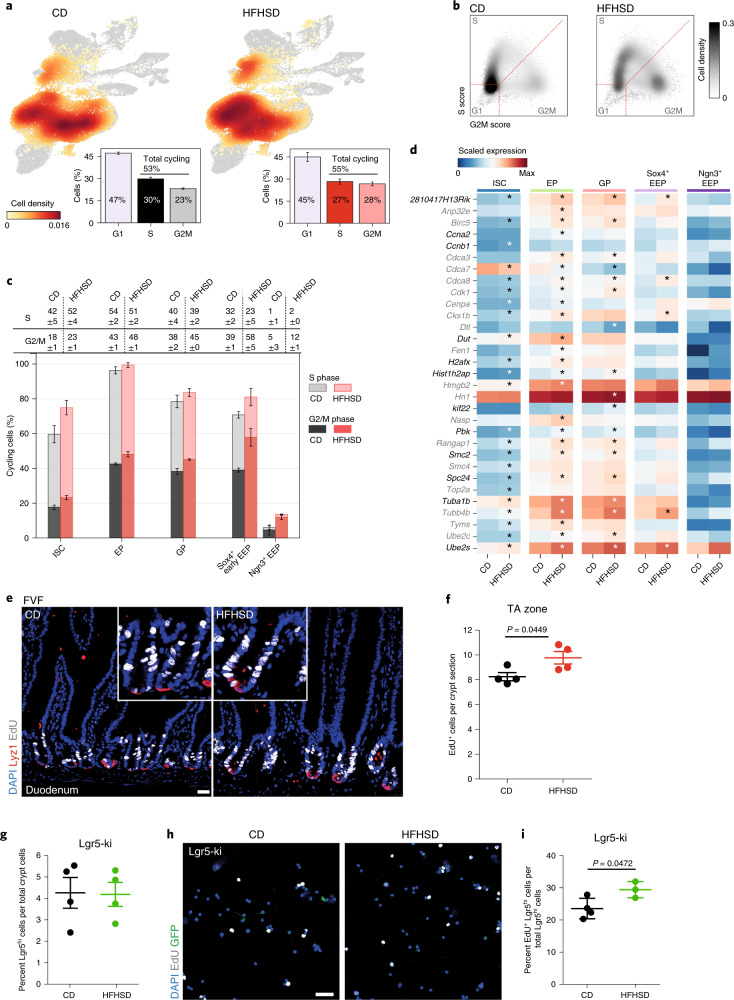
HFHSD induces hyperproliferation of ISCs and progenitors. **a**, Distribution of cycling cells across CD- and HFHSD-derived cell clusters depicted as cell densities projected onto the UMAP plot and quantified as proportions of cells in each cell-cycle stage. Cells were classified using a cell-cycle score, calculated using the expression of genes related to cell cycle. Data are mean ± s.e.m. of biologically independent samples, *n* = 3 mice per group. **b**, Distribution of ISCs and progenitors over the three cell-cycle stages visualized as cell densities in a scatter-plot of S- versus G2/M-phase score levels. Higher score levels indicate higher expression of involved genes. Dotted lines depict classification borders. Densities are Gaussian kernel estimates. **c**, Proportions of cycling cells in CD and HFHSD-derived ISCs and progenitor clusters. Table indicates percentages. Data are shown as mean ± s.e.m. of biologically independent samples, *n* = 3 mice per group. EP, enterocyte progenitor; GP, goblet progenitor. **d**, Heatmap of mean expression values per cluster of selected genes used for cell-cycle scoring (black) or highly correlating with S and G2M scores (grey, Pearson correlation >0.7). Only cells classified as cycling (S or G2M phase) are shown. * Indicates differentially expressed genes between CD and HFHSD conditions (two-sided, limma, adjusted *P* < 0.01, logFC > 0.1), *n* = 3 mice per group. FC, fold change. *P* values are provided in Supplementary Table 5. **e**,**f**, Representative LSM images (**e**) and quantification (**f**) of EdU incorporation after a 2-h EdU (white) pulse in the TA zone in duodenal sections of CD- and HFHSD FVF mice. Lyz1^+^ Paneth cells are shown in red. Scale bar, 25 µm, *n* = 4 mice per group. Data are mean ± s.e.m. of biologically independent samples. Statistical significance was determined by two-tailed Student’s *t*-test. FDR, false discovery rate. **g**, Determination of Lgr5–EGFP^hi^ cells from CD- and HFHSD-fed Lgr5-ki mice by flow cytometry, *n* = 4 mice per group. Data are mean ± s.e.m. of biologically independent samples. **h**,**i**, Representative LSM images from cytospin (**h**) and quantification (**i**) of EdU^+^Lgr5–EGFP^hi^ cells from CD- and HFHSD-fed Lgr5-ki mice after a 2-h EdU (white) pulse. Scale bar, 40 µm, *n* = 4 CD mice, *n* = 3 HFHSD mice. Data are mean ± s.d. of biologically independent samples. Statistical significance was determined by two-tailed Student’s *t*-test. Source data

**Extended Data Fig. 8:**
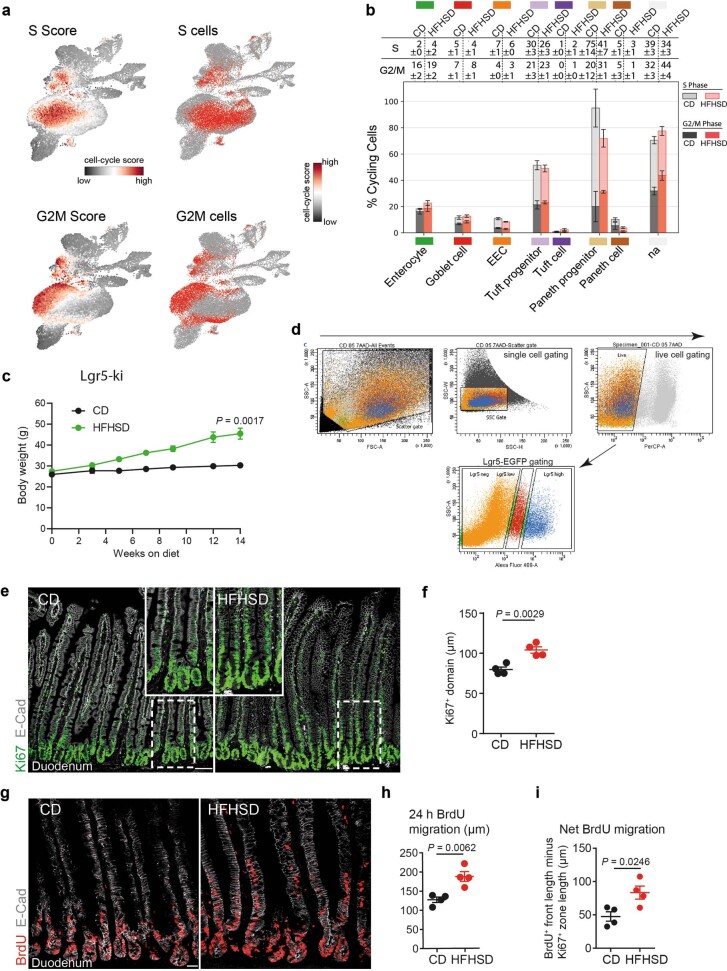
HFHSD increases turnover of SI crypt cells. **a**, Left: Cell score levels for cell-cycle phases S and G2/M calculated based on the expression of a set of genes related to cell-cycle are visualized in a UMAP plot. Right: Cells assigned to the corresponding cell-cycle phase are highlighted in red. **b**, Proportions of cycling cells assigned to S and G2/M cell-cycle phases in CD and HFHSD-derived in indicated clusters. Table indicates percentages. Data are shown as mean ± s.e.m., *n* = 3 mice per group. **c**, Assessment of body weight over the course of 14 weeks of CD- and HFHSD-fed Lgr5-ki mice. *n* = 4 per group. Data are shown as mean ± s.e.m. of biologically independent samples. Statistical significance was determined by two-tailed Student’s t-test. **d**, Representative FACS plots showing gating strategy to determine the proportion of Lgr5-EGFP^hi^ cells (ISCs) in CD and HFHSD fed Lgr5-ki mice. **e**, **f**, Representative LSM images (**e**) and quantification (**f**) of Ki67^+^ (green) proliferative regions. Scale bar, 75 µm, *n* = 4 mice per group. Data are mean ± s.e.m. of biologically independent samples. Statistical significance was determined by two-tailed Student’s t-test. **g**–**i**, Representative LSM images of BrdU-labelled cells (BrdU^+^ nuclei, red) along the crypt-villus axis 24 h after BrdU administration in duodenal sections from CD- and HFHSD-fed FVF mice (**g**). Quantification of cell migration length by measuring the distance from the crypt base to the highest labelled cell in the villus (**h**). Net BrdU migration (**i**), calculated as the average distance of BrdU-labelled migration front minus the Ki67^+^ zone to correct for increased cell proliferation on HFHSD. Scale bar, 25 µm. *n* = 4 mice per group. Data are mean ± s.e.m. of biologically independent samples. Statistical significance was determined by two-tailed Student’s t-test. Source data

We next asked whether the increased number of Ngn3^+^ EEPs, as well as the increase in villus length and higher abundance of the enterocyte and goblet cell lineage under HFHSD conditions, resulted from enhanced proliferation. To test this, we identified proliferating cells using a cell-cycle signature score that assigns each cell to a cell-cycle state (G1, S and G2M; Methods) (Extended Data Fig. 8a). HFHSD mainly increased the proportion of cells in G2M phase, indicating that HFHSD alters cell-cycle dynamics (Fig. 4a,b). In particular, in ISCs, enterocytes, goblet cells and the early *Sox4*^+^ and *Ngn3*^+^ EEP clusters, the proportion of cycling cells was clearly increased (Fig. 4c and Extended Data Fig. 8b). Moreover, several genes, including the known cell-cycle regulators *Ccnb1*, *Cenpa*, *Dut*, *Pbk* and *Smc2*, which correlated with the S/G2M cell-cycle score, were differentially expressed in proliferating cells upon HFHSD, further suggesting that cell-cycle dynamics of ISCs and progenitors is accelerated (Fig. 4d and Supplementary Table 5).

To confirm the increase in proliferative activity, we first identified 5-ethynyl-2ʹ-deoxyuridine (EdU)-positive progenitor cells 2 h after an EdU pulse. Consistent with the crypt scRNA-seq data, we found a higher number of proliferative progenitors in the transit-amplifying (TA) zone in SI crypts of HFHSD-fed FVF mice (Fig. 4e,f). To directly assess the number of proliferating ISCs we put Lgr5-ki mice^33^ on an HFHSD (Extended Data Fig. 8c). In contrast to our scRNA-seq data, we found no difference in the numbers of ISCs between CD and HFHSD by flow cytometry on the basis of high Lgr5-EGFP fluorescence intensity (Extended Data Fig. 8d and Fig. 4g). However, the numbers of EdU-positive ISCs were increased on HFHSD (Fig. 4h,i).

Further, we determined antigen immunoreactivity to Ki67 and the migration rate of crypt cells. We observed that Ki67-labeled domains were significantly larger in mice fed an HFHSD and, in contrast to controls, extended into the villi. Also, the crypt-to-villus migration rate of pulse-labeled 5-bromo-2ʹ-deoxyuridine (BrdU)-positive cells was increased on HFHSD (Extended Data Fig. 8e–i) which altogether suggests that an HFHSD enhances cell turnover.

In summary, these data demonstrate that an obesogenic diet increases the proliferation rate of ISCs and progenitors leading to increased villus length. Enhanced cell-cycle activity of ISCs did not result in physical expansion of the ISC pool due to accelerated differentiation and cell turnover. This is reflected by the decreased fraction of ISCs and increased fraction of progenitors in our crypt scRNA-seq data (Fig. 1e). These results further indicate that ISC homeostasis and identity is disturbed in HFHSD-fed animals.

### HFHSD upregulates fatty acid synthesis and Ppar signalling

**Extended Data Fig. 9:**
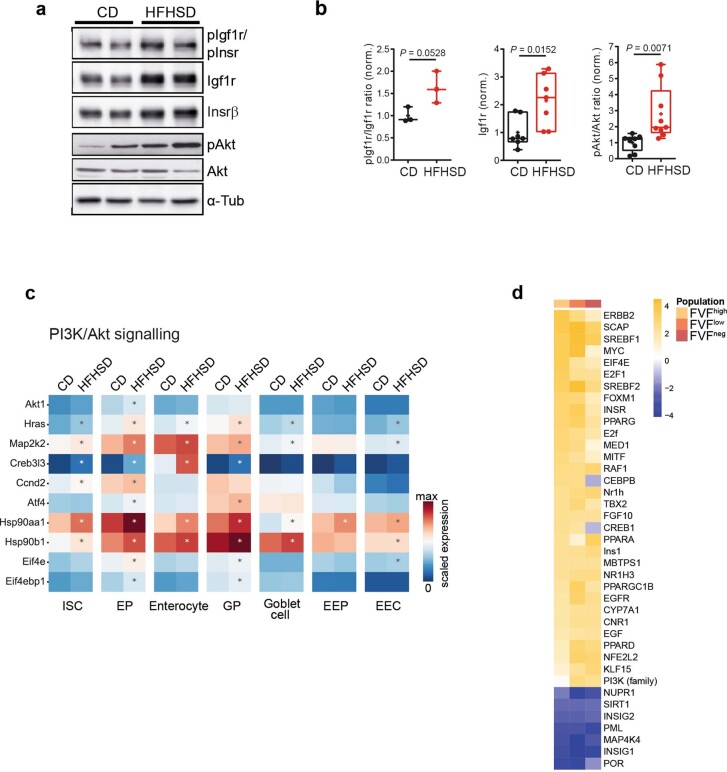
HFHSD stimulates the Insr/Igf1r/Akt pathway and fatty acid synthesis and Ppar signaling. **a**, **b**, Representative immunoblots of phosphorylated (p)Igf1r/Insr (Tyr1158/Tyr1162/Tyr1163) and total Igf1r, Insrβ, pAkt (Ser473) and total Akt (**a**) and quantification of the protein levels (**b**). Protein expression was analysed in whole-cell lysates from SI crypts of CD- and HFHSD-fed FVF animals. For quantification, signal intensities of all protein bands were normalised to α-tubulin; phosphorylated proteins were additionally normalised to the corresponding non-phosphorylated protein. *n* = 3 mice per group for pIgf1r/pInsr, *n* = 7 mice per group for Igf1r, *n* = 9 mice per group for (p)Akt and total Akt. Data are presented as box-and-whisker plots. The lower and upper boundaries of the boxes represent the 25th and 75th percentiles, respectively. The center lines indicate the medians, the crosses denote the mean values, and whiskers represent the maximum and minimum values. Statistical significance was determined by two-tailed Student’s t-test. Circles represent biological independent samples. **c**, Mean expression levels per cluster of genes involved in PI3K/Akt signaling in CD and HFHSD. * indicates a significant change in HFHSD mice (limma, FDR < 0.01, logFC>0.1)). The p-values are provided in Supplementary table 1. **d**, Predicted activation or inhibition of upstream regulators in bulk transcriptomics. Significantly regulated genes from each population (each HFHSD versus CD) were analysed by Ingenuity Pathway Software to predict the activation (yellow; z-score >2) or inhibition (blue; z-score < −2) of upstream regulators. Shown are all genes with three z-scores available and from which at least two were significant (z-score > ׀2 ׀). Source data

A central role of metabolic pathways in the regulation of stem cell maintenance and fate control has been described in several adult stem cell systems^34,35^. Both Wnt/β-catenin and Igf1/insulin signalling integrate metabolic and proliferative cues, and increased activity of these pathways has been shown to induce ISC hyperproliferation upon high-fat diet (HFD) feeding^6,36–38^. We assessed Wnt/β-catenin pathway activation in bulk ISC-enriched FVF^low^ cells and FVF^neg^ enterocyte progenitors, which we isolated by flow cytometry (Supplementary Fig. 2a,b). Unexpectedly, the level of nuclear β-catenin was decreased or unchanged in ISC-enriched FVF^low^ cells or enterocyte progenitor-enriched FVF^neg^ cells, respectively (Supplementary Fig. 2c,d). Consistently, expression of Wnt/β-catenin target genes was downregulated in ISC-enriched FVF^low^ cells and HFHSD-derived single-cell ISCs (Supplementary Fig. 2e–g and Supplementary Table 1). As glycogen synthase kinase 3β (Gsk3β) is a negative regulator of Wnt/β-catenin signalling and an important modulator of cellular metabolism, we checked Gsk3β activity^39^. Decreased levels of phosphorylated Gsk3β in HFHSD-derived SI crypts indicated increased Gsk3β activity and enhanced Gsk3β-mediated destruction of β-catenin (Supplementary Fig. 2h,i). Together, these results demonstrate that Wnt/β-catenin signalling does not drive hyperproliferation of ISCs and progenitors and enhanced progenitor recruitment in our HFHSD mouse model. Instead, decreased Wnt/β-catenin signalling indicates that differentiation of ISCs is accelerated and thus provides further evidence of disturbed ISC homeostasis^40,41^. HFHSD caused obesity and hyperinsulinaemia in our mouse model. Analysis of InsrIgf1rAkt pathway activity in SI crypt lysates showed increased phosphorylation of InsrIgf1r and Akt as well as upregulation of several genes associated with PI3K and Akt signalling, which is in line with pronounced hyperinsulinaemia and confirms previous findings that Igf1insulin signalling induces hyperproliferation in diet-induced obesity (Extended Data Figs. 1r–u and 9a–c).

**Fig. 5:**
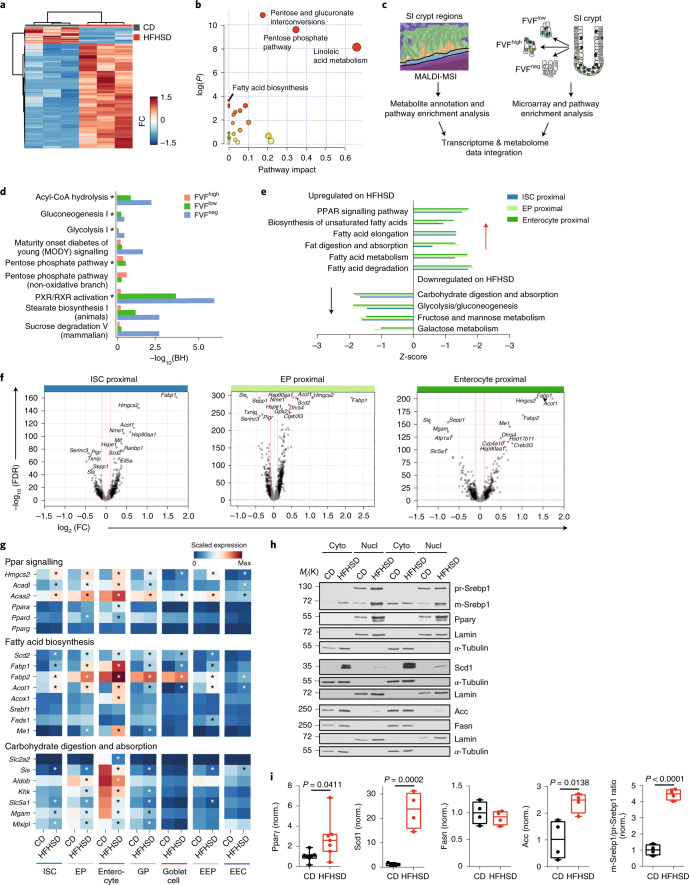
Fatty acid synthesis and Ppar signalling are upregulated on HFHSD. **a**, Heatmap-based clustering analysis of the 297 discriminative metabolite masses (FC ≥ 2 and *P* ≤ 0.05). Each coloured cell on the map corresponds to an intensity value, with samples in rows and features in columns. Euclidean distance and Ward’s method were applied for clustering analysis. Statistical significance was determined by two-tailed Student’s *t*-test. **b**, Pathway enrichment analysis of deregulated metabolites was performed with MetaboAnalyst 3.0. The enrichment method was hypergeometric test. Topology analysis was based on relative betweenness centrality. The *P* value was calculated from the enrichment analysis without adjustment (FC ≥ 2 and *P* ≤ 0.05). Metabolic pathways are represented as circles according to their scores from enrichment (vertical axis) and topology analyses (pathway impact, horizontal axis). **c**, Overview of experimental design for data integration. **d**, Ingenuity pathway analysis showing overlap of significantly deregulated metabolites on HFHSD from MALDI–MSI profiling and genes from microarray analysis. Shown are values from microarray analysis and the red line indicates the significance cutoff. **e**, Enriched KEGG pathways in genes differentially regulated between CD and HFHSD conditions (Enrichr, Fisher’s exact test, two-sided). Genes with FDR < 0.01 and logFC > 0.1 were considered and weighted by logFC. **f**, Volcano plots showing differential expression and its significance (−log_10_(FDR), limma) for each gene on HFHSD compared to CD. Red lines indicate thresholds used for significance level and gene expression change and regulated genes are highlighted in black. Annotated genes are the top ten genes ranked by FDR. **g**, Mean expression levels for selected genes. * Indicates a significant change (limma, FDR < 0.01, logFC > 0.1). **h**,**i**, Protein expression analysis by western blot in cytoplasmic (cyto) and nuclear (nucl) extracts from SI crypts of CD- and HFHSD-fed FVF mice. Representative immunoblots (**h**) and relative quantification of band signal intensity (**i**), *n* = 4 mice per group (Srebp1, Scd1, Acc, Fasn) and *n* = 7 mice per group (Pparγ). Data are presented as box-and-whisker plots. The lower and upper boundaries of the boxes represent the 25th and 75th percentiles, respectively. The centre lines indicate the medians and whiskers represent the maximum and minimum values. Statistical significance was determined by two-tailed Student’s *t*-test. Circles represent biological independent samples. norm., normalized. Source data

**Extended Data Fig. 10:**
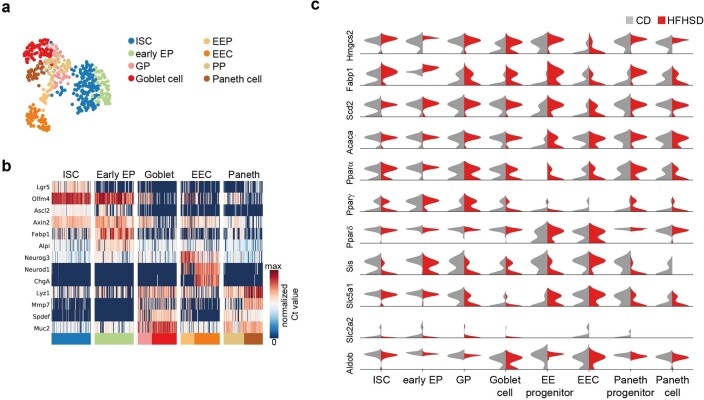
Confirmation of expression differences in metabolic genes in FVF-positive cells from CD- and HFHSD-fed mice by targeted single-cell qPCR. **a**, **b**, UMAP plot of 465 profiled FVF positive crypt cells from 3 CD and 3 HFHSD-fed mice coloured by cell type (**a**). Cluster annotation was based on lineage marker gene expression depicted in the heatmap (**b**). **c**, Violin plots showing expression of selected metabolic genes related to fatty acid synthesis, Ppar signaling and glycolysis in indicated single-cell clusters split by diet.

To uncover additional metabolic pathways that link HFHSD to hyperproliferation and cell fate changes, we compared metabolite profiles from SI crypt regions of CD- and HFHSD-fed FVF mice using matrix-assisted laser desorption/ionization mass spectrometry imaging (MALDI–MSI)^42^. MALDI–MSI allows analysis of metabolites directly in tissue sections without isolation bias. We identified 297 discriminative masses; of these, 257 were enriched and 40 were less abundant in SI crypts of HFHSD-fed animals (Fig. 5a). Pathway enrichment analysis revealed that HFHSD upregulated metabolite signatures related to fatty acid biosynthesis (for example, octadecanoic acid) and linoleic acid metabolism (for example, phosphatidylcholine) and downregulated metabolites linked to pathways of glucose metabolism, such as the pentose phosphate pathway and pentose glucuronate interconversions (for example, d-glyceraldehyde 3-phosphate) (Fig. 5b and Supplementary Table 6). To map the metabolic changes from tissue to cell-type level, we integrated metabolomics data with bulk transcriptomes of ISC-enriched FVF^low^ cells, secretory progenitor-enriched FVF^high^ cells and enterocyte progenitor-enriched FVF^neg^ cells (Extended Data Fig. 9d, Fig. 5c and Methods). The HFHSD metabolite signature overlapped with regulated genes involved in carbohydrate metabolism (for example, sucrose degradation, maturity onset diabetes of young (MODY) signalling) and pro-proliferative fatty acid biosynthesis pathways (for example, stearate synthesis and acyl-CoA-hydrolysis)^43^. Regulation of these pathways was most pronounced in enterocyte progenitor-enriched FVF^neg^ and ISC-enriched FVF^low^ cells (Fig. 5d and Supplementary Table 7). Next, to determine the cell subtypes in which an HFHSD altered metabolism and to identify the molecular pathways associated with deregulated HFHSD metabolites, we compared the transcriptional profiles of CD- and HFHSD-derived cells. We found that gene signatures associated with Ppar signalling (for example *Hmgcs2*, *Acdvl*, *Acaa2*, *Fabp1* and *Fabp2*) and fatty acid biosynthesis (for example *Acot1*, *Acox1*, *Scd2*, *Srebf1*, *Fads1* and *Me1*) were upregulated in HFHSD-derived ISCs and progenitor clusters (Fig. 5e–g and Supplementary Tables 1,8 and 9). Upregulation of these gene sets was stronger in subpopulations with proximal identity; in particular, in proximal ISCs, enterocyte and goblet progenitors (Fig. 5e,f and Supplementary Table 8). Moreover, genes associated with carbohydrate metabolism (for example glycolysis, gluconeogenesis and the pentose phosphate pathway) were downregulated in these subtypes (Fig. 5e–g). We confirmed upregulation of active Srebp1 (mature, m-Srebp1), Acc, Pparγ and Scd1, the master transcriptional regulators and key enzymes of Ppar signalling and fatty acid synthesis in HFHSD crypt protein lysates and in ISCs and progenitors, by a sensitive, targeted single-cell qPCR approach (Fig. 5h,i, Extended Data Fig. 10 and Supplementary Table 10). Finally, consistent with crypt enterocytes, we also found that mature villus enterocytes increase the expression of genes associated with intracellular fat accumulation and lipid uptake, de novo lipogenesis and peroxisomal fatty acid oxidation (Supplementary Fig. 3).

Thus, by integration of in situ metabolomics, bulk and single-cell transcriptomics and targeted protein expression analysis, we have revealed diet-induced metabolic rewiring and cell- and subtype-specific transcriptional changes that correlate with an increase in proliferation and endocrine dysfunction.

## Discussion

In this study we provide a basic mechanistic explanation of diet-induced ISC dysfunction and intestinal maladaptation that underlie the development of obesity and prediabetes and increase the risk for gastrointestinal cancer.

With our study we aimed to determine the immediate effects of a western-style HFHSD on intestinal function. We observed enlargement of the SI, longer villi and decreased crypt density and an altered cellular composition in the crypts, which was confirmed in the villi by lineage-tracing studies. We are aware that our lineage-tracing approach using the dual-fluorescent, inducible Cre-reporter *Foxa2*^nEGFP-CreERT2/+^;*Gt(ROSA)26*^mTmG/+^ mouse has its limitations; for example, inefficient labelling of stem cells, which generates fewer clonal ribbons in comparison to the Lgr5-ki reporter^33^ and analysis of only one time point. Therefore, we validated our findings from the crypt scRNA-seq experiment not only by a lineage-tracing approach but also independent of a reporter in tissue sections.

We show that ISCs and progenitors are hyperproliferative, and that differentiation and cell turnover are accelerated by an HFHSD. Accelerated differentiation and cell turnover also explain the discrepancy between the scRNA-seq data (decrease in ISCs) and the data from the Lgr5-reporter mice (no change in the number of ISCs). ISCs in HFHSD-fed mice divide faster and newly formed cells are still Lgr5–EGFP^hi^, but already express markers of differentiated cells. Whole-transcriptome-based clustering in our scRNA-seq data groups Lgr5^hi^ ISCs and Lgr5^hi^ progenitors separately, whereas when using only Lgr5–EGFP as a marker in tissue sections and flow cytometry we cannot discriminate between an ISC and a progenitor.

Hyperproliferation in the crypt, which can promote cancer initiation and progression, has previously been associated with an HFD; however, the molecular pathways that couple dietary cues to this cellular response are still debated and probably depend on the type of diet (for example fat and sugar source)^37,38,44^. Also, a role of metabolic pathways in ISC maintenance, number and fate control has been highlighted in several studies^7,44,45^. Hyperproliferation upon HFHSD is not driven by Pparδ-mediated activation of the major oncogenic pathway in the gut, Wnt/β-catenin signalling, as previously reported for a lard-based chronic HFD^6,46,47^. Instead, we found that a coconut oil- and sucrose-based high-lipid and -carbohydrate content diet specifically elevates pro-proliferative Pparγ signalling, Srebp1-mediated lipogenesis and InsrIgf1r–Akt signalling. Upregulation of Pparγ and Srebp1-mediated de novo lipogenesis has been associated with inflammation, increased proliferation and tumour progression in many types of cancer and thus provides a possible link between HFHSD-induced metabolic signalling, crypt-cell hyperproliferation and increased risk of gastrointestinal cancer^43^. As tumour initiation and progression require biomass production and therefore depend on a high nutrient supply, chronic activation of these pathways as observed with an HFHSD might reduce the barrier to oncogenic transformation or tumour growth and proliferation.

Further, we found a profound impact of an HFHSD on the enterocyte lineage. Enterocytes are metabolically rewired, increase the expression of genes linked to carbohydrate and fat uptake and show an intracellular fat accumulation, which is reminiscent of the vesicular accumulation of triglycerides in hepatic steatosis that causes liver fibrosis and cancer^48–50^. In addition, HFHSD induces a regional and spatial repatterning of enterocyte gene expression and function. An HFHSD increased the number of proximal-type enterocytes, which are specialized on carbohydrate and fatty acid absorption and altered enterocyte zonation along the crypt–villus axis (increased expression of the fatty acid transporter Fabp1). Thus, our results imply that enterocytes functionally adapt to an HFHSD, which may increase calorie intake and fat accumulation and promote obesity.

Our in-depth molecular and functional analysis of the EEC lineage reveals mechanisms of EEC dysfunction in obesity that include (1) higher abundance of Ngn3^+^ EEPs owing to increased proliferation, (2) lower abundance of serotonergic *Reg4*^+^ EC cells and lower plasma serotonin levels, (3) higher abundance of peptidergic Ghrl^+^ SILA cells but lower ghrelin plasma levels, (4) increased numbers of ileal Gcg^+^ cells and increased Glp-1 levels and (5) lower abundance of *Lgr5*^+^ EECs. The physiological roles of gut-derived serotonin are broad and it regulates various processes both in the gut and systemically^32^. Accumulating evidence indicates a link between peripheral serotonin and systemic glucose and lipid metabolism, as well as metabolic diseases^51^. Whether blood serotonin levels are changed in obesity is controversial, due to difficulties in measuring serotonin^51^. However, intraperitoneal injection of 5-HT to mice inhibits weight gain, hyperglycaemia and insulin resistance on an HFD^52^. Thus, reduced numbers of serotonin-producing cells and blood serotonin levels as observed in our mouse model might promote the development of obesity. The hunger hormone ghrelin is known to increase gastric emptying and decrease insulin secretion^53^. A negative correlation between plasma insulin and ghrelin has been reported in human obesity, which is in line with our data^54^. Notably, we found that lower plasma ghrelin levels are not due to a reduced number of Ghrl^+^ SILA cells, suggesting that an impaired secretory machinery might affect ghrelin levels. However, given that ghrelin-secreting cells are also present in the stomach, changes in plasma ghrelin levels may not be solely attributed to duodenal Ghrl^+^ EECs. Lower postprandial GLP-1 levels are reported in obesity and incretin hormone secretion and activity are impaired in individuals with prediabetes, although findings are contradictory^9,55^. In our obesity model, however, the number of Gcg-expressing cells and Glp-1 plasma levels were increased. Glp-1 stimulates insulin secretion from pancreatic β-cells, so the higher levels of circulating Glp-1 in HFHSD-fed mice may be a compensatory response to insulin resistance at the prediabetic state, which leads to hyperinsulinaemia.

Finally, in contrast to other studies, Paneth cell numbers were not affected in our obesity model^6^.

T﻿he discrepancies between our results and those of previous studies support the emerging evidence that the intestine fine-tunes its response to environmental stimuli^6,37,38^. Different factors, such as the selected mouse model (for example, diet versus genetically induced obesity), type of diet (for example HFD versus HFHSD), fat source (for example lard versus coconut oil) and/or duration of diet (short-term versus chronic) can influence intestinal remodelling and response. For instance, in contrast to the coconut oil, high-sucrose diet for 12–14 weeks used in this study, a lard-based low-sugar chronic diet for 9–14 months caused shorter villi, increased the number of ISCs, decreased the number of Paneth cells and did not affect goblet cells and EECs^6^. Hence, careful selection and reporting of dietary information in animal studies is crucial to interpret and contextualize results.

In summary, our study reveals that functional maladaptation of the gut in response to an HFHSD is caused by disturbed ISC identity, changes in the regional identity of cells and an altered mature cell-type composition. Further, we describe targetable pathways that are induced by an HFHSD and potentially underlie the pathogenesis of the metabolic syndrome and gastrointestinal cancer. This new understanding of the mechanisms of disease is crucial to develop non-invasive therapeutic options to resolve obesity and insulin-dependent diabetes, for example by counteracting enteroendocrine dysregulation (for example, by elevating peripheral serotonin levels) and increased nutrient absorption (for example, by inducing distal enterocyte phenotypes).

## Methods

### Experimental model

#### Animals

Animal experiments were carried out in compliance with the German Animal Protection Act and with the approved guidelines of the Society of Laboratory Animals and of the Federation of Laboratory Animal Science Associations. This study was approved by the institutional Animal Welfare Officer (Helmholtz Center Munich) and by the Government of Upper Bavaria, Germany. Homozygous FVF mice were generated as previously described and backcrossed to C57BL/6 background^22^. *Foxa2*^nEGFP-CreERT2^ mice^25^ (CD1 background) were crossed with *Gt(ROSA)26*^mTmG^ mice^24^ (mixed 129/SvJ, C57BL/6J background) to obtain heterozygous *Foxa2*^nEGFP-CreERT2/+^*;Gt(ROSA)26*^mTmG/+^ animals and bred in our own facilities. Other mouse lines were *Lgr5-EGFP-IRES-creERT2* (ref. ^33^) (Lgr5-ki, C57BL/6J background) and wild-type C57BL/6N (bred in our own facilities).

Mice were housed in groups of two to four animals and maintained at 23 ± 1 °C and 45–65% humidity on a 12-h dark/light cycle with ad libitum access to diet and water unless otherwise indicated. All experiments were performed using male animals at 3 to 7 months of age.

### Dietary interventions

For dietary interventions, 10–12-week-old male mice were randomized into test groups matched for body weight, with similar variance, and given ad libitum access to either an obesogenic HFHSD (58% kcal from fat, 25% kcal from carbohydrates, 17% kcal from protein (Research Diets, no. D12331)) or CD (11% kcal from fat, 64% kcal from carbohydrates, 25% kcal from protein (ssniff Spezialdiäten, E15051-04)) for a period of 11–13 weeks. Body weights were measured every second week.

### Body composition analysis

Lean and fat masses were measured in FVF mice 12 weeks after the start of the diet using quantitative nuclear magnetic resonance technology (EchoMRI).

### Glucose tolerance and insulin secretion tests

Glucose tolerance was assessed by an oral glucose tolerance test (oGTT) in FVF mice, maintained for 12 weeks on CD or HFHSD. After a 6-h fast, mice received an oral glucose bolus (1.5 mg g^−1^ body weight of 20 % (*wt*/*v*) d-(+)-glucose (Sigma-Aldrich) in PBS). Tail blood glucose concentrations were measured with a handheld glucometer (Abbott) before (0 min) and 15, 30, 60 and 120 min after the glucose bolus. To measure the insulin secretion, tail vein blood samples were collected into EDTA-coated microvette tubes (SARSTEDT) at time points 0, 15 and 30 min of the oGTT. Plasma was extracted by centrifugation (3,500 r.p.m., 15 min, 4 °C) and insulin concentration was determined using the Ultra-Sensitive Mouse Insulin ELISA Kit (Crystal Chem, 90080) according to the manufacturer’s instructions.

The homeostasis model assessment of insulin resistance (HOMA-IR) and HOMA-β were used to assess insulin resistance and beta-cell function, respectively, in FVF mice at 12 weeks after diet start. HOMA indices were calculated from basal blood glucose and plasma insulin levels after a 6-h fast based on the conventional formulas: HOMA-IR = fasting blood glucose (mg per 100 ml) × fasting insulin (µU per ml)/405 and HOMA-β = fasting insulin (µU per ml) × 360/fasting glucose (mg per 100 ml) − 63 (ref. ^56^).

### Plasma hormone measurements

Circulating hormones, serotonin, ghrelin and Glp-1 were assessed in FVF mice maintained for 13 weeks on a CD or an HFHSD. For basal levels (ghrelin and serotonin), mice were fasted for 6 h and tail vein blood was sampled into EDTA-coated microvette tubes (SARSTEDT). To compare postprandial plasma hormone levels (Glp-1, ghrelin), fasted mice were gavaged with 250 µl of mixed-meal-containing liquid diet (Osmolite HiCal, Abbott) supplemented with dextrose at 20% (*wt*/*v*) (Sigma-Aldrich). Blood was collected 10 min after the mixed-meal bolus either from the tail vein or, under terminal anaesthesia with isoflurane, from the vena cava^57–59^. For Glp-1 measurement, blood samples were immediately mixed with 0.1 mM Diprotin A (Abcam, 145599) and 500 KIU ml^−1^ aprotinin (Sigma, A-1153). Plasma was extracted by centrifugation (13,000 r.p.m., 2 min, 4 °C). Total ghrelin concentrations were determined using an ELISA kit from Millipore-Merck (EZRGRT-91K), total plasma Glp-1 was measured using a mouse Glp-1 ELISA kit (Crystal Chem, 81508) and serotonin concentration was determined using a serotonin ELISA kit from Enzo (ADI-900-175).

### Proliferative cell labelling with EdU and BrdU and tamoxifen administration in mice

To assess the epithelial replication rate in the SI, EdU (Thermo Fisher Scientific, A10044) or BrdU (Sigma, no. B5002) was administered as an intraperitoneal injection at 100 μg g^−1^ body weight or at 50 μg g^−1^ body weight, respectively, each from a 10 mg ml^−1^ stock and in sterile PBS. Mice were killed 2 h post-EdU or 24 h post-BrdU administration. For the assessment of BrdU label retention, FVF mice maintained for 10 weeks on a diet were given BrdU in drinking water at 1 mg ml^−1^ supplemented with 1% sucrose for 14 d. BrdU was then withdrawn and mice were further maintained on a diet with ad libitum drinking water for a chase period of 21 d. A group of mice was killed after 14 d of continuous BrdU labelling or after a 21d period of chase. For short-term genetic lineage studies, *Foxa2*^nEGFP-CreERT2/+^*;Gt(ROSA)26*^mTmG/+^ mice were fasted for 3 h and Cre-recombinase activity was induced by a titrated single dose of tamoxifen administered orally by gavage (Sigma-Aldrich, T5648) at 0.25 mg g^−1^ body weight in sunflower oil. Mice were killed 70 h after the tamoxifen gavage.

### Crypt and villus isolation and single-cell preparation

Isolation of small intestinal crypts was carried out as previously reported^60^. In brief, SIs were removed and washed with cold PBS. Villi were scraped off with a glass slide. The remaining tissue was cut into 2-cm pieces, washed several times with cold PBS and incubated in 2 mM EDTA/PBS for 35 min at 4 °C on a tube roller. Subsequently, crypts were collected by rigorous shaking and filtered through a 70-µm mesh to remove villous fragments. For single-cell preparation, isolated crypts were incubated with TrypLE (Life Technologies, no. 12605) for 5 min on ice and then 5 min at 37 °C and treated with 10 µg ml^−1^ DNase in crypt complete medium (DMEM/F-12 containing 10% FCS) for 5 min at 37 °C. Single-cell suspension was achieved by gentle repeated pipetting. Cells were washed twice with 2% FCS in PBS and pelleted at 300*g* for 5 min at 4 °C. For flow cytometry, cells were collected in 1–2 ml FACS buffer (2% FCS, 2 mM EDTA in PBS) (Sigma-Aldrich, no. Y0503) and passed through 40-µm cell strainer caps of FACS tubes.

To obtain a single-cell suspension of villi cells, villi were scraped off and incubated with TrypLE as described for crypt cells.

### Flow cytometry

For gene expression measurement (microarray, single-cell transcriptomics) and western blotting, small intestinal crypt cells were sorted using FACS-Aria III (FACSDiva software v.6.1.3, BD Bioscience) with a 100-µm nozzle. For all experiments, single cells were gated according to their FSC-A (front scatter area) and SSC-A (side scatter area). Singlets were gated dependent on the FSC-W (front scatter width) and FSC-H (front scatter height) and dead cells were excluded using 7-AAD (eBioscience, no. 00-6993-50). For quantitative PCR with reverse transcription (qRT–PCR), cells were sorted directly into Qiazol lysis reagent (QIAGEN, no. 79306). To obtain FVF-enriched small intestinal crypt-cell samples for scRNA-seq, we sorted 30,000 FVF^+^ (FVF^low^ and FVF^high^) cells followed by sorting 30,000 live crypt cells per sample. Cells were sorted into modified FACS buffer (2% FCS, 0.02 mM EDTA in PBS).

### RNA isolation for qRT–PCR and microarray

For bulk gene profiling studies (qPCR, microarray), RNA isolation from crypts or flow-sorted cells was performed using the RNA isolation kit miRNeasy Mini (QIAGEN, no. 217004) or miRNeasy Micro kit (QIAGEN, no. 217084) depending on the amount of the sample. Complementary DNA was synthesized using the SuperScript VILO cDNA synthesis kit (Invitrogen, no. 11754). RNA was reverse transcribed and amplified with the Ovation PicoSL WTA System V2 kit (NuGEN, no. 331248).

### Microarray analysis

For gene profiling of flow-sorted FVF^low^, FVF^high^ and FVF^neg^ cells from CD- and HFHSD-fed FVF mice, total RNA was isolated as described above and RNA integrity was assessed using an Agilent 2100 Bioanalyzer (Agilent RNA 6000 Pico Kit). RNA was amplified with the Ovation PicoSL WTA System V2 in combination with the Encore Biotin Module (Nugen). Amplified cDNA was hybridized on Affymetrix Mouse Gene 1.0 ST arrays. Staining (Fluidics script FS450_0007) and scanning of the microarray were performed according to the Affymetrix expression protocol including minor modifications as suggested in the Encore Biotin kit protocol. Expression Console (v.1.3.0.187, Affymetrix) was used for quality control and annotation of the normalized robust microarray analysis gene-level data, and standard settings, including median polish and sketch-quantile normalization, were used.

### TaqMan qRT–PCR

For gene expression analysis, real-time qRT–PCR was performed using TaqMan probes (Life Technologies), TaqMan Fast Advanced Master Mix (Applied Biosystems, no. 4444557) or TaqMan Universal Master Mix II (Applied Biosystems, no. 4440040) for amplified cDNA and the ViiA 7 Real-Time PCR System (Thermo Fisher Scientific).

The following probes were used: Mm00782745_s1 for *Rpl37*, Mm00438890_m1 for *Lgr5*, Mm01320260_m1 for *Olfm4*, Mm01268891_g1 for *Ascl2*, Mm00443610_m1 for *Axin2*, Mm03928990_g1 for *RN18S*, Mm02524776_s1 for *Fzd2*, and Mm00433409_s1 for *Fzd7*.

### scRNA-seq: RNA preparation, library generation and sequencing

FVF-enriched single-cell samples of crypts isolated from the small intestinal epithelium (duodenum, jejunum and ileum) and villus samples from the SI of C57BL/6N mice were prepared as described above. Dead cells were excluded by flow cytometry after 7AAD labelling. Dead cell exclusion was controlled by trypan blue staining and sorted cells were counted. Single-cell libraries were generated using the Chromium Single cell 3′ library and gel bead kit v2 (10X Genomics, no. 120237) according to the manufacturer’s instructions. Libraries were sequenced on a HiSeq4000 (Illumina) with 150-bp paired-end sequencing of read 2.

### MALDI–MSI

Fresh-frozen samples were cut into 12-μm sections using a cryo-microtome at −20 °C (Leica CM1950, Leica Microsystems) and mounted onto precooled conductive indium-tin-oxide-coated MALDI target glass slides (Bruker Daltonics). Sections were coated with 9-aminoacridine hydrochloride monohydrate matrix (Sigma-Aldrich) at 10 mg ml^−1^ in water/methanol 30:70 (*v*/*v*) by a SunCollect automatic sprayer (Sunchrom). The matrix application was performed at flow rates of 10, 20 and 30, respectively, for the first three layers. The other five layers were performed at 40 μl min^−1^. MALDI–MSI measurement was performed on a Bruker Solarix 7T FT-ICR-MS (Bruker Daltonics). MALDI–MSI data were acquired over a mass range of *m*/*z* 50–1,000 in negative ionization mode with 30-μm spatial resolution using 50 laser shots at a frequency of 500 Hz. The acquired data underwent spectrum processing in FlexImaging v.4.2 (Bruker Daltonics). Following MALDI imaging experiments, the matrix was removed with 70% ethanol. Tissue sections were stained with haematoxylin and eosin. Slides were scanned with a MIRAX DESK digital slide-scanning system (Carl Zeiss MicroImaging).

### Western blot

For protein expression analyses, whole-cell lysates from isolated crypts or flow-sorted cells were prepared using the RIPA buffer (50 mM Tris, pH 7.5, 150 mM NaCl, 1 mM EDTA, 1% Igepal, 0.1% SDS, 0.5% sodium deoxycholate) containing phosphatase (Sigma-Aldrich, P5726, P0044) and proteinase inhibitors (Sigma-Aldrich, P8340). Nuclear and cytosolic extracts from isolated crypts were prepared using the NE-PER Nuclear and Cytoplasmic Extraction Reagents kit (Thermo Fisher Scientific, no. 78833) according to the manufacturer’s instructions. Cell lysates were mixed with Laemmli sample buffer, resolved by SDS–PAGE and blotted onto a PVDF membrane (Bio-Rad). Membranes were blocked with 5% milk in Tris-buffered saline containing 0.2% Tween-20, then incubated overnight with primary antibodies in blocking solution at 4 °C, followed by a 1-h incubation with horseradish peroxidase (HRP)-conjugated IgG secondary antibodies. Protein bands were visualized using a chemiluminiscence reagent (Bio-Rad, no. 170-5061) and quantified using ImageJ software. For quantification, expression of proteins was normalized to α-tubulin or lamin in cytoplasmic or nuclear fractions, respectively.

Primary antibodies used were mouse anti-Srebp1 (1:1,000 dilution, Novus Biologicals, NB600-582SS); rabbit anti-Acc (1:1,000 dilution, Cell Signaling Technology, 3676); rabbit anti-Pparγ (1:1,000 dilution, Cell Signaling Technology, 2435); goat anti-lamin (1:1,000 dilution, Santa Cruz, sc-6217); mouse anti-α-tubulin (1:1,000 dilution, Sigma-Aldrich, T6199); rabbit anti-Fasn (1:1,000 dilution, Cell Signaling Technology, 3180); rabbit anti-Scd1 (1:1,000 dilution, Cell Signaling Technology, 2794); mouse anti-β-catenin (1:1,000 dilution, BD, 610154), rabbit anti-Gsk3β (1:5,000 dilution, Cell Signaling Technology, 12456); and rabbit anti-phospho Gsk3β (1:5,000 dilution, Cell Signaling Technology, 5558). Secondary antibodies used were goat anti-mouse HRP (1:15,000 dilution, Dianova, 115036062); goat anti-rabbit HRP (1:15,000 dilution, Dianova, 111036045) or rabbit anti-goat HRP (1:15,000 dilution, Dianova, 305035045).

### Tissue morphology assessment

For tissue histology, intestines were flushed and fixed in 4% paraformaldehyde (PFA) overnight, paraffin embedded according to standard procedures and sectioned at 6 µm. Sections were dried, dehydrated through a graded ethanol series and cleared in xylene. Standard haematoxylin and eosin staining was performed and images were acquired using the Zeiss AXIO Scope A1 microscope (Carl Zeiss AG).

### Histochemistry and immunofluorescence

SIs were isolated, rinsed with ice-cold PBS and fixed with 4% PFA for 3 h at 4 °C. Tissue was cryopreserved through a progressive sucrose gradient (7.5% for 1 h, 15% for 1 h, 30% sucrose overnight), embedded in a tissue-freezing medium (Leica Biosystems, no. 14020108926) and sectioned at 14 µm. For whole-mount staining, isolated small intestinal crypts were fixed in 4% PFA for 30 min at room temperature (RT) and subsequently washed in PBS. For immunofluorescence staining, sections or crypts were permeabilized with 0.5% Triton X-100 in PBS for 30 min at RT, blocked (10% FCS, 0.1% BSA and 3% donkey serum in PBS/0.1% Tween-20) for 1 h and incubated with primary antibodies overnight at 4 °C. Sections or crypts were washed in PBS/0.1% Tween-20 and incubated with secondary antibodies in blocking solution for 1 h at RT, stained with DAPI (ROTH, 6335.1) to visualize the nuclei and mounted with the Elvanol antifade reagent.

To assess proliferation, EdU staining was performed using the Click-iT Staining kit (Invitrogen, no. C10340) according to the manufacturer’s instructions.

For BrdU staining, sections were incubated with 3.3 N HCl for 10 min on ice, followed by incubation for 50 min at 37 °C and two wash steps with borate buffer, pH 8.5, to neutralize the reaction (each wash 15 min at RT).

Ki67 immunoreactivity was assessed on paraffin tissue sections after heat-induced antigen retrieval (at ~90 °C, 10 min) using the Antigen Unmasking Solution, Citric Acid-Based (Vector Laboratories, no. H-3300-250).

Fluorescent images were obtained with a Leica SP5 confocal microscope (Leica Microsystems) and analysed using LAS AF software (LAS AF software v.2.6.0-7266).

The primary antibodies used were chicken anti-GFP (1:600 dilution, Aves Labs, GFP-1020); goat anti-ChgA (1:200 dilution, Santa Cruz, sc-1488); rabbit anti-Lyz1 (1:1,000 dilution, DAKO, M0776); rabbit anti-Muc2 (1:1,000 dilution, Santa Cruz, sc-7314); rat anti-BrdU (1:200 dilution, Abcam, ab6326); rabbit anti-5-HT (1:1,000 dilution, Neuromics, RA20080); anti-rabbit Ngn3 (1:100 dilution, a kind gift from H. Edlund); goat anti-villin (1:200 dilution, Santa Cruz, sc-7672); goat anti-ghrelin (1:200 dilution, Santa Cruz, sc-10368); rabbit anti-Ki67 (1:200 dilution, Abcam, ab15580); rabbit anti-E-cadherin (extracellular domain) (1:1,000 dilution, a gift from D. Vestweber); rabbit anti-Fabp1 (1:300 dilution, Abcam, ab222517); and rabbit anti-Apoa4 (1:300 dilution, Abcam, ab231660). The secondary antibodies used were donkey anti-chicken Alexa Fluor 488 (1:800 dilution, Dianova, 703225155); donkey anti-mouse Cy5 (1:800 dilution, Dianova, 715175151); donkey anti-goat Alexa Fluor 555 (1:800 dilution, Invitrogen, A21432); donkey anti-rabbit Alexa Fluor 555 (1:800 dilution, Invitrogen, A31572); and donkey anti-rabbit Alexa Fluor 649 (1:800 dilution, Dianova, 711605152).

### RNAScope in situ hybridization for detection of target RNA

In situ detection of *FOXA2*, *LGR5* and *OLFM4* mRNA was performed using the RNAscope Intro Pack for Multiplex Fluorescent Reagent Kit v2-Mm (ACD; no. 323136) according to the manufacturer’s protocol. RNAscope 3-Plex Negative Control Probe (dapB; ACD; no. 320871) and RNAscope 3-Plex Positive Control Probe (ACD; no. 320881) were used as internal controls. Probes used were Mm-Foxa2 (ACD; no. 409111), Mm-Olfm4-C2 (ACD; no. 311831-C2) and Mm-Lgr5-C3 (ACD; no. 312171-C3) with respective fluorescent dyes Opal 520 Reagent (AKOYA; no. FP1487001KT) for channel 1, Opal 570 Reagent (AKOYA; no. FP1488001KT) for channel 2 and Opal 690 Reagent (AKOYA; no. FP1497001KT) for channel 3, diluted 1:800 in RNAscope Multiplex TSA Buffer (ACD; no. 322809). The assay was performed on FFPE mouse intestinal jejunum sections prepared as described above with a thickness of 7 µm and standard pretreatment conditions with protease III (ACD; no. 322340) as recommended by the manufacturer.

After completion of the RNAscope assay, sections were stained using DAPI (ROTH, 6335.1) to visualize the nuclei, washed with PBS three times and mounted with ProLong Diamond Antifade Mountant (Life Technologies; no. P36970). Samples were visualized using a Leica SP5 confocal microscope (LAS AF software v.2.6.0-7266).

### Gene expression analysis from bulk sorted cells

Statistical bulk transcriptome analyses were performed using the statistical programming environment R implemented in CARMAweb^61,62^. Gene-wise testing for differential expression was carried out employing the limma *t*-test. Sets of regulated genes were defined by raw *P* < 0.01, FC > 1.3× and average expression in at least one group >32. Enriched canonical pathways were analysed through the use of QIAGEN’s ingenuity pathway analysis (https://www.qiagen.com/ingenuity). Microarray data are available at Gene Expression Omnibus (GEO).

### Bioinformatics and statistical analysis of MALDI–MSI data

MATLAB R2014b (v.7.10.0, Mathworks) was used as MALDI spectral pre-processing tool for the subsequent data bioinformatics analysis. Peak picking was performed using an adapted version of the LIMPIC algorithm^63^. In brief, the parameters of peak picking included *m*/*z* 0.0005 minimal peak width, signal-to-noise threshold of 4 and intensity threshold of 0.01%. Isotopes were automatically identified and excluded. Statistical comparisons were performed with a Student’s *t*-test (two-tailed). Metabolites were considered to be significant if they had an intensity FC ≥ 2 and a *P* value ≤0.05. Metabolite annotation was performed by matching accurate mass with databases (mass accuracy ≤4 ppm, METLIN, http://metlin.scripps.edu/; Human Metabolome Database, http://www.hmdb.ca/; MassTRIX, http://masstrix3.helmholtz-muenchen.de/masstrix3/; METASPACE, http://annotate.metaspace2020.eu/). Heat-map-based clustering and enrichment analysis of metabolic pathways were performed with MetaboAnalyst v.3.0 (http://www.metaboanalyst.ca).

### Integration of bulk transcriptome and metabolome

Lists of genes participating in the candidate pathways from the ingenuity canonical pathways analysis of the microarray data of FVF^low^, FVF^high^ and FVF^neg^ cells were compiled. For each of the genes on the lists, information on the reaction it is involved in and the participating metabolites were extracted from the mouse specific BiGG databank (https://www.ncbi.nlm.nih.gov/pmc/articles/PMC2874806/, https://www.ncbi.nlm.nih.gov/pubmed/20959003). If genes and metabolites within a reaction were also significant in their analyses as described above, the reaction and its pathway were said to be affected at both the metabolomics and the transcriptomics levels.

### Single-cell gene expression analysis by microfluidic qRT–PCR

To assess the transcriptional profiles of single FVF^low^ and FVF^high^ cells, a nested single-cell qPCR design was used. FVF^low^ or FVF^high^ cells isolated from three CD- and HFHSD-fed FVF mice were sorted as described above directly into single wells of 96-well plates containing 5 μl of a pre-amplification solution composed of 1.2 μl 5× VILO reaction mix (Invitrogen, no. 11754-050), 0.3 μl 20 U μl^−1^ SUPERase-In (Ambion, no. AM2694), 0.25 μl 10 % NP40 (Thermo Fisher Scientific, no. 28324), 0.25 μl RNA spikes mix (Fluidigm, no. 100-5582) and 3 μl of nuclease-free water (Promega, no. P119C). Cells were lysed by incubation at 65 °C for 90 s and cDNA transcription from RNA was performed by reverse transcription cycling (25 °C for 5 min, 50 °C for 30 min, 55 °C for 25 min, 60 °C for 5 min and 70 °C for 10 min) with 1 μl reverse transcription mix solution containing 0.15 μl 10× SuperScript enzyme mix (Invitrogen, no. 11754-050), 0.12 μl T4 Gene 32 Protein (New England BioLabs, no. M0300S) and 0.73 μl nuclease-free water. The efficiency and specificity of outer and inner primer pairs for target-specific cDNA amplification were tested in advance. Primers showing single peaks and single bands by melt curve analysis and by separation of qPCR products on a 2.5% agarose gel, respectively, were considered specific. Primer efficiency was analysed over a range of tenfold cDNA dilutions (1:1 to 1:100) and primers with 100 ± 15 % efficiency were qualified for further proceedings. Specific target amplification was performed with 9 μl reaction mix containing 7.5 μl TaqMan PreAmp Master Mix (Applied Biosystems, no. 4391128), 0.075 μl 0.5 M EDTA, pH 8.0 (Invitrogen, no. Am9260G), 1.5 μl 10× outer primer mix (500 nM) under the following cycling conditions: enzyme activation step at 95 °C for 10 min, 20 cycles of denaturation for 5 s at 96 °C and 4 min annealing/extension at 60 °C. Amplified cDNA samples were then treated with 6 μl Exonuclease I reaction mix containing 0.6 μl reaction buffer, 1.2 μl Exonuclease I (New England BioLabs, no. M0293S) and 4.2 μl nuclease-free water. To increase target specificity, amplified single-cell cDNA samples were analysed with gene-specific inner primer pairs and SsoFast EvaGreen Supermix with Low ROX (Bio-Rad Laboratories, no. 172-5210) using the 96.96 Dynamic Array on the BioMark System (Fluidigm). BioMark Real-Time PCR Analysis software (Fluidigm) was used to calculate Ct values.

### Computational analyses of single-cell data

A detailed description of the computational analyses of single-cell data is provided in the Supplementary information.

### Statistical analyses

Data collection was performed using Microsoft office Excel 2016–2018 and statistical analysis was performed using GraphPad Prism 6 Software (GraphPad Software). All data are shown as mean ± s.e.m. unless otherwise specified. In box-and-whiskers plots, data are represented as minimum and maximum with centre lines indicating the median. All samples represent biological replicates. For statistical significance testing of two independent groups, an unpaired two-tailed Student’s *t*-test was used. For statistical comparison of longitudinal data (body weight curves, GTT and IST), two-way analysis of variance corrected by Sidak’s multiple comparison test was used. *P* values of < 0.05 % were considered statistically significant. For metabolic studies (GTT and IST), sample size was statistically determined; otherwise sample size estimates were not used. Studies were not blinded and investigators were not blinded during analysis.

### Reporting Summary

Further information on research design is available in the Nature Research Reporting Summary linked to this article.

## Supplementary information

Supplementary Information Supplementary Figs. 1–5, Supplementary Table 6 and Methods (Computational analyses of single-cell data).

Reporting Summary

Supplementary Table 1 Differentially expressed genes in major intestinal crypt populations in HFHSD mice. Differentially expressed genes between CD and HFHSD conditions in major intestinal crypt populations. Differential expression between conditions was performed using limma-trend (Methods). Genes with FDR < 0.01, logFC > 0.1 were included in the table.

Supplementary Table 2 State-specific genes of endocrine lineage formation in intestinal crypt cells. Endocrine state-specific genes capturing transient gene expression during endocrine lineage formation in intestinal crypt cells. State markers were identified by pairwise differential expression testing of each subpopulation against all other subpopulations using a Wilcoxon rank-sum test. Genes with a test-score > 5 and within top 1,200 ranking genes in every test were considered as state markers.

Supplementary Table 3 Lineage-specific genes of endocrine lineage formation in intestinal crypt cells. Endocrine lineage-specific genes capturing expression in progenitor cells of endocrine lineages in intestinal crypt cells. Lineage markers were identified by pairwise differential expression testing of each endocrine progenitor populations against all other progenitors except for progenitors of a later stage using a Wilcoxon rank-sum test. Genes with a test-score > 5 and within top 1,200 ranking genes in every test were considered as lineage markers.

Supplementary Table 4 Differentially expressed genes in crypt EEC subpopulations in HFHSD mice. Differentially expressed genes between CD and HFHSD conditions in crypt EEC subpopulation. Differential expression between conditions was performed using limma-trend (Methods). Genes with FDR < 0.01, logFC > 0.1 were included in the table.

Supplementary Table 5 Differentially expressed genes in cycling cells of major intestinal crypt populations in HFHSD mice. Differentially expressed genes between CD and HFHSD conditions in cycling cells of major intestinal crypt populations. Differential expression between conditions was performed using limma-trend (Methods). Genes with FDR < 0.01, logFC > 0.1 were included in the table.

Supplementary Table 7 Cell-type specific integration of bulk transcriptome and metabolome data shows overlap between regulated genes and metabolites. Lists of genes participating in the candidate pathways from the ingenuity canonical pathways analysis of the microarray data of FVF^low^, FVF^high^ and FVF^neg^ cells were compiled. For each of the genes on the lists, information on the reaction it is involved in and the participating metabolites were extracted from the mouse specific BiGG databank (https://www.ncbi.nlm.nih.gov/pmc/articles/PMC2874806/ and https://www.ncbi.nlm.nih.gov/pubmed/20959003). If genes and metabolites within a reaction were also significant in their analyses as described above, the reaction and its pathway were said to be affected on both the metabolomics and the transcriptomics levels.

Supplementary Table 8 Differentially expressed genes in major intestinal crypt populations split by gut region in HFHSD mice. Differentially expressed genes between CD and HFHSD conditions in major intestinal crypt populations split by gut region. Differential expression between conditions was performed using limma-trend (Methods). Genes with FDR < 0.01, logFC > 0.1 were included in the table.

Supplementary Table 9 Pathway enrichment of differentially expressed genes in major intestinal crypt populations split by gut region in HFHSD mice. Enriched KEGG pathways in differentially expressed genes between CD and HFHSD conditions in major intestinal crypt populations split by gut region. Pathway enrichment was performed using Enrichr and a Fisher’s exact test. Genes with FDR < 0.01, logFC > 0.1 were considered and weighted by their logFC (Methods).

Supplementary Table 10 Cell numbers in CD and HFHSD conditions measured in targeted single-cell qPCR approach.

Supplementary Data 1 Source data for Supplementary Fig. 2.

## Data Availability

All data generated or analysed during this study are included in this article and its supplementary files. Microarray data have been submitted to NCBI/GEO (GSE148227). scRNA-seq data have been submitted to NCBI/GEO (GSE147319). Source data are provided with this paper.

## Source data

Source Data Fig. 1 Statistical source data.
Source Data Fig. 2 Statistical source data.
Source Data Fig. 3 Statistical source data.
Source Data Fig. 4 Statistical source data.
Source Data Fig. 5 Statistical source data.
Source Data Fig. 5 Unprocessed western blot.
Source Data Extended Data Fig. 1 Statistical source data.
Source Data Extended Data Fig. 4 Statistical source data.
Source Data Extended Data Fig. 6 Statistical source data.
Source Data Extended Data Fig. 7 Statistical source data.
Source Data Extended Data Fig. 8 Statistical source data.
Source Data Extended Data Fig. 9 Statistical source data.
Source Data Extended Data Fig. 9 Statistical source data for unprocessed western blot.

## Code availability

Code and custom scripts for scRNA-seq analysis have been deposited on https://github.com/theislab/2021_Aliluev_Tritschler_gut_HFD.
