# Supplementary material for: Diet-induced alteration of intestinal stem cell function underlies obesity and prediabetes in mice
Source: Nat Metab. 2021 Sep 22;3(9):1202–16. doi: 10.1038/s42255-021-00458-9 (PMC8458097; doi:10.1038/s42255-021-00458-9)
Supplement: Supplementary file 1 — Supplementary Figs. 1–5, Supplementary Table 6 and Methods (Computational analyses of single-cell data). [file 42255_2021_458_MOESM1_ESM.pdf]

nature portfolio

https://doi.org/10.1038/s42255-021-00458-9

Supplementary information

Diet-induced alteration of intestinal stem cell function underlies obesity and prediabetes in mice

In the format provided by the authors and unedited

nature portfolio

https://doi.org/10.1038/s42255-021-00458-9

Supplementary information

Diet-induced alteration of intestinal stem cell function underlies obesity and prediabetes in mice

In the format provided by the authors and unedited

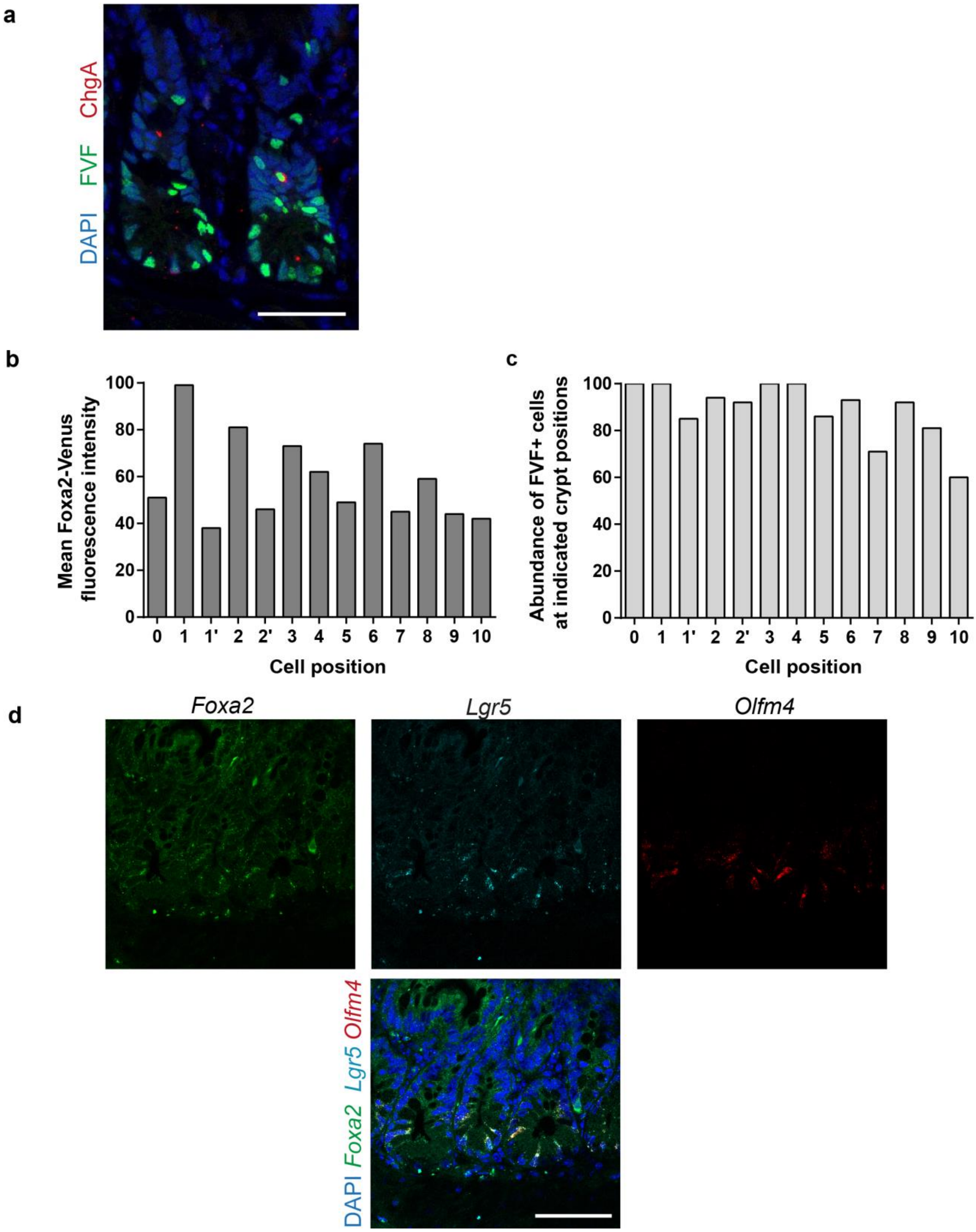
a
DAPI FVF ChgA
b
Mean Foxa2-Venus fluorescence intensity
100
80
60
40
20
0
0 1 1' 2 2' 3 4 5 6 7 8 9 10
Cell position
c
Abundance of FVF+ cells at indicated crypt positions
100
80
60
40
20
0
0 1 1' 2 2' 3 4 5 6 7 8 9 10
Cell position
d
Foxa2
Lgr5
Olfm4
DAPI Foxa2 Lgr5 Olfm4

Supplementary Figure 1: Foxa2 is expressed in quiescent and rapidly cycling ISCs.

a, Representative image of jejunal tissue sections from FVF reporter mice showing FVF reporter expression in the crypt. Scale bar, 20µm.

b, c, Quantification of the mean Foxa2-Venus fluorescence intensity (b) and frequency of FVF-positive cells (c) at indicated crypt positions. N=42 crypts. Scale bar, 20µm.

d, Representative confocal image showing the co-expression of *Foxa2*, *Olfm4* and *Lgr5* mRNA in duodenal crypt sections by RNAscope. Scale bar, 20µm.

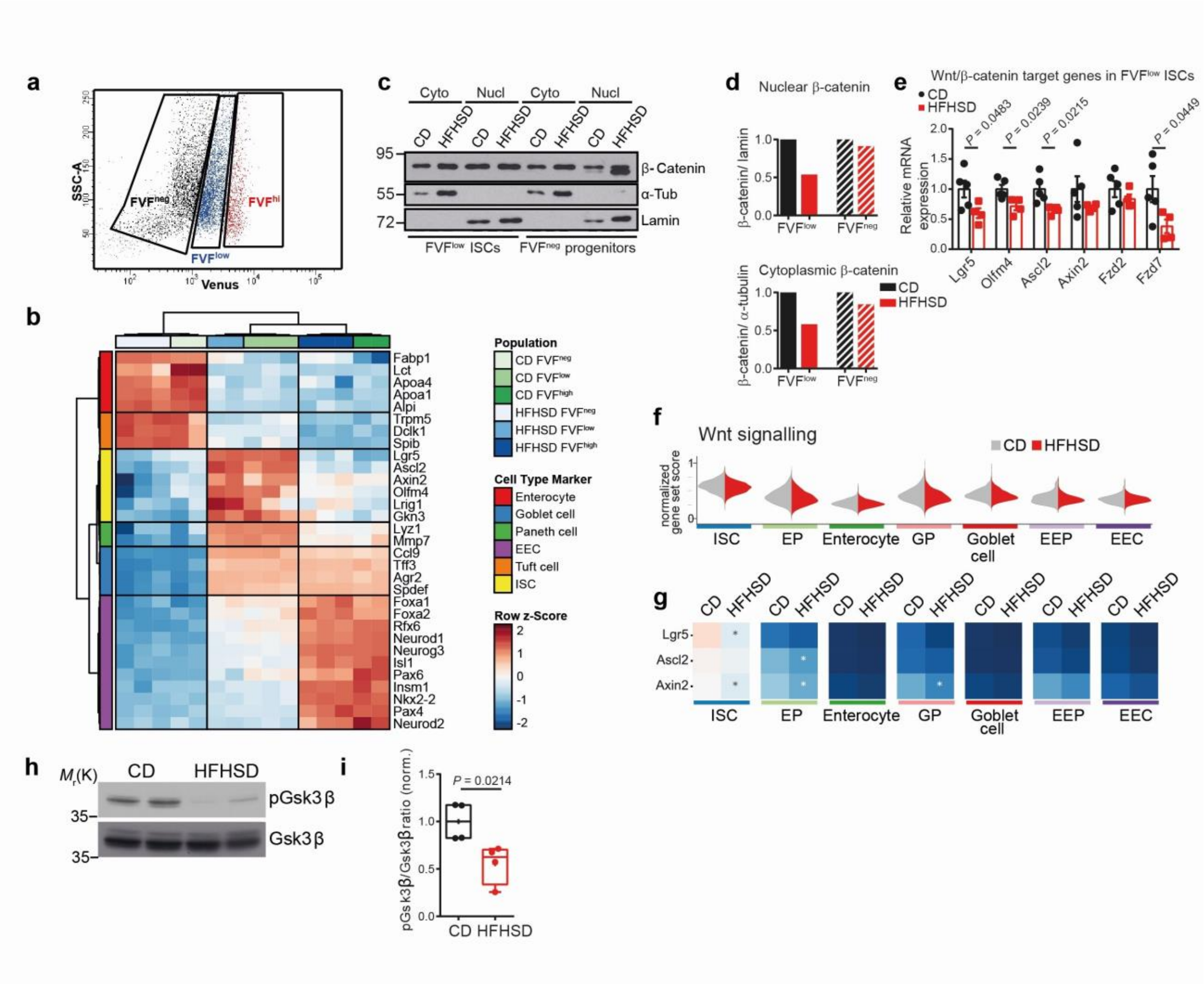

Supplementary Figure 2: HFHSD does not activate Wnt/β-catenin signalling.

a, Flow cytometry gating strategy for gene expression analysis of FVF^{high}, FVF^{low} and FVF^{neg} SI crypt cells derived from CD- and HFHSD-fed FVF animals. Initial gating was set on doublet-exclusion followed by gating for live cells.

b, Heatmap showing relative expression levels (row-wise Z scores) of key intestinal lineage marker genes in FVF^{high}, FVF^{low} and FVF^{neg} cells from microarray analysis.

c, d Western blot analysis (c) and quantification (d) of β-catenin levels in cytoplasmic and nuclear extracts from flow sorted FVF^{low} and FVF^{neg} SI crypt cells of CD- and HFHSD-fed FVF mice. For quantification, β-catenin expression was normalised to α-tubulin or lamin in cytoplasmic or

nuclear protein extracts, respectively. One sample per group, each sample represents pooled cells from 4 mice.

e, Gene expression analysis of representative ISC marker and β-catenin target genes in flow sorted FVF^{low} (enriched for ISCs) cells from CD- and HFHSD-fed FVF mice by qRT-PCR. Results were normalised to multiple housekeeping genes (18S, Rpl37) and expressed as means of fold change relative to the control ±SEM of biologically independent samples. Statistical significance was determined by two-tailed Student's t-test. n=5 mice versus 4 mice per group.

f, Wnt signalling score distribution in indicated single-cell clusters split by diet visualized using violin plots. Wnt signalling score was calculated based on the expression of a gene set involved in Wnt signalling (see Methods).

g, Mean expression levels per cluster of ISC marker genes, *Lgr5*, *Ascl2* and *Axin2* in CD and HFHSD. * indicates a significant change in HFHSD mice (limma, FDR <0.01, logFC>0.1).

h, i, Representative immunoblots of phosphorylated (p) Gsk3β (Ser9) and total Gsk3β and quantification of the protein levels (i). Protein expression was analysed in whole-cell lysates from SI crypts of CD- and HFHSD fed FVF animals. For quantification, signal intensities of all protein bands were normalised to α-tubulin; phosphorylated proteins were additionally normalised to the corresponding non-phosphorylated protein. n=4 per group. Data are presented as box-and-whisker plots. The lower and upper boundaries of the boxes represent the 25th and 75th percentiles, respectively. The centre lines indicate the medians, the crosses denote the mean values, and whiskers represent the maximum and minimum values. Statistical significance was determined by two-tailed Student's t-test. Circles represent biological independent samples.

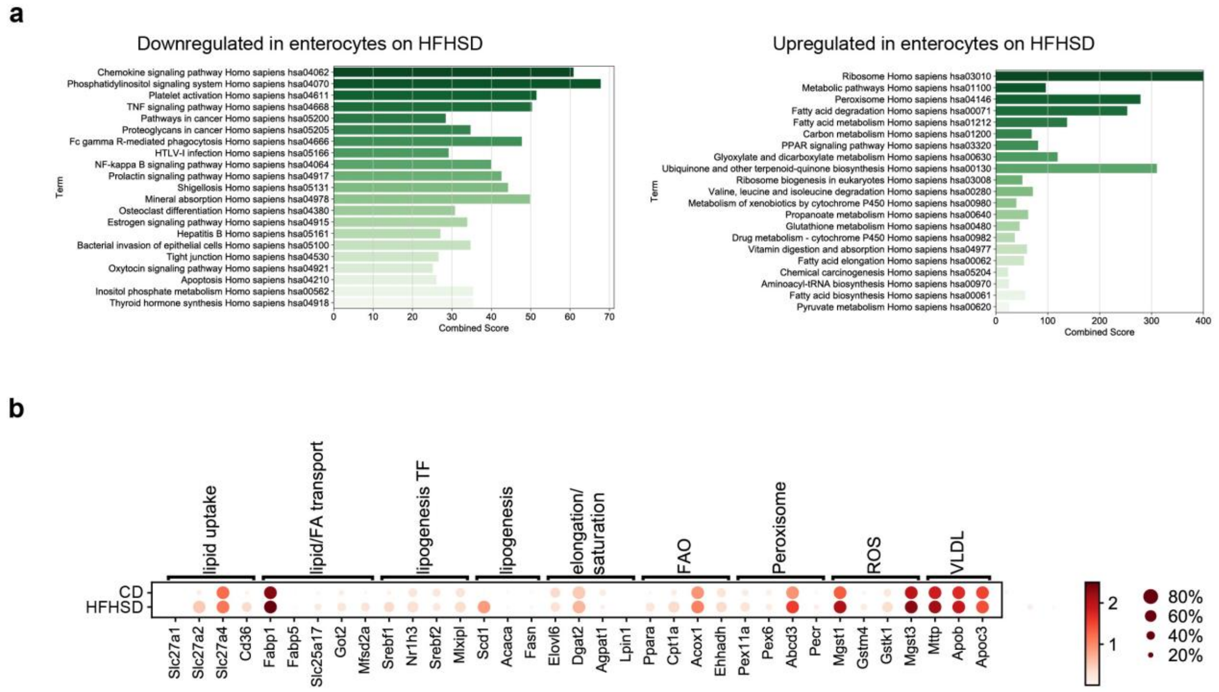

Supplementary Figure 3: Enterocytes functionally adapt to a HFHSD.

a, Enriched KEGG pathways in genes differentially regulated between CD and HFHSD enterocytes.

b, Dot plot showing the differential expression of selected functional genes in HFHSD vs CD enterocytes.

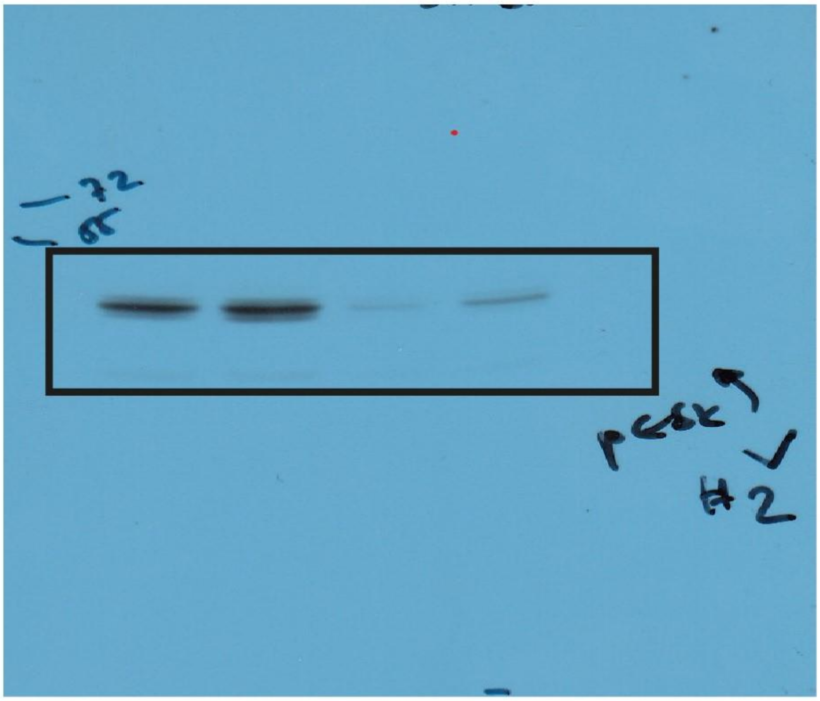

pGsk3 beta

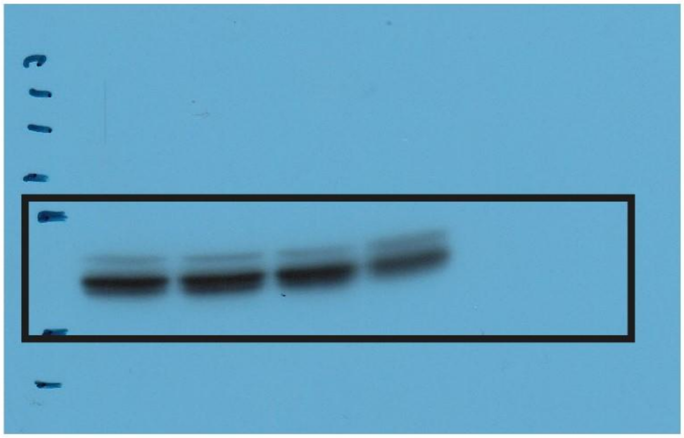

Gsk3 beta

Supplementary Figure 4: Unprocessed western blot of Supplementary Figure 2h (source data).

	Discriminative pathways	p-value	Components
**Enhanced in HFHSD**	Linoleic acid metabolism	0.0003	C00157 Phosphatidylcholine<br>C00219 Arachidonate<br>C01595 Linoleate<br>C03242 (8Z,11Z,14Z)-Icosatrienoic acid<br>C04056 9-cis,11-trans-Octadecadienoate<br>C06426 (6Z,9Z,12Z)-Octadecatrienoic acid<br>C07289 Crepenynate
**Enhanced in HFHSD**	Fatty acid biosynthesis	0.0256	C00712 (9Z)-Octadecenoic acid<br>C01530 Octadecanoic acid<br>C02679 Dodecanoic acid<br>C06424 Tetradecanoic acid<br>C08362 (9Z)-Hexadecenoic acid
**Reduced in HFHSD**	Pentose phosphate pathway	<0.0001	C00117 D-Ribose 5-phosphate<br>C00118 D-Glyceraldehyde 3-phosphate<br>C00199 D-Ribulose 5-phosphate<br>C00231 D-Xylulose 5-phosphate<br>C00620 alpha-D-Ribose 1-phosphate<br>C01236 D-Glucono-1,5-lactone 6-phosphate<br>C04442 2-Dehydro-3-deoxy-6-phospho-D-gluconate
**Reduced in HFHSD**	Pentose and glucuronate interconversions	<0.0001	C00111 Glycerone phosphate<br>C00199 D-Ribulose 5-phosphate<br>C00231 D-Xylulose 5-phosphate<br>C01101 L-Ribulose 5-phosphate<br>C03291 L-Xylulose 5-phosphate<br>C06441 L-Xylulose 1-phosphate

Supplementary Figure 5: Discriminative pathways distinguish HFHSD and CD (Supplementary table 6).

Table shows pathway enrichment analysis for metabolites identified by MALDI-MSI.

Computational analyses of single-cell data

Preprocessing of droplet-based scRNA-seq data

Demultiplexing of raw base call (BCL) files, alignment, read filtering, barcode and UMI counting were performed using the CellRanger analysis pipeline (Version 2.0.0) provided by 10X Genomics. Reads were aligned to the mm10-reference genome downloaded from the 10X-Website. All further analyses were run using the python-based Scanpy API except stated otherwise (for software specifications and code availability see below)[63]. For selection of high quality cell barcodes first the standard CellRanger cell detection algorithm was used which is based solely on the UMI count distribution. Since a high variability of RNA content and number of genes expressed per cell (UMI count > 1) among cell types was observed a further selection step was performed and cells with more than 1000 genes expressed were additionally included. We further filtered cells with a high fraction of counts from mitochondrial genes (10% or more), since this is commonly interpreted as a sign for stressed or dying cells. Cell by gene count matrices of all samples were then concatenated to a single data matrix and values log transformed (log(count+1)). Genes with expression in less than 20 cells were excluded. To facilitate clustering batch correction was performed using a python implementation of ComBat available here https://github.com/brentp/combat.py with default parameters and specifying each sample as one batch[64]. ComBat was recommended by an extensive batch correction method comparison and evaluation[65]. To identify contaminating non-epithelial cells an initial round of unsupervised graph-based clustering (louvain) was performed. A clearly distinct cluster of cells highly expressing immune cell marker genes was removed. Selection of top 2000 variable genes was performed based on normalized dispersion of genes as previously described using the pp.filter_genes_dispersion function from the Scanpy API with default parameters. Changing the number of selected top variable genes to 1500 or 3000 did not influence further analyses much (data not shown). This output matrix was used as an input for all further analysis except for differential expression testing where log transformed raw data was used without batch correction and instead batch was included as covariate (see below).

Low dimensional embedding and clustering (cell type annotation)

The single cell neighborhood graph was computed on the 50 first principal components using Scanpy (pp.neighbors) with a local neighborhood size of 25 and a Gaussian kernel for computing connectivities (method=gauss). Uniform Manifold Approximation and Projection (UMAP) was run for visualization as recently recommended[66]. To identify cell subtypes louvain-based clustering at varying resolution in different parts of the data manifold was used as adopted by Scanpy from louvain-igraph (Traag V., 2017, https://github.com/vtraag/louvain-igraph) (‚split-

and-merge' approach, for detailed steps of clustering analysis see code). Cell subtypes of all lineages were annotated based on the expression of known marker genes and clusters merged if only reflecting heterogeneity within a subtype. Novel marker genes and characteristic gene signatures were identified using the tl.rank_genes function from Scanpy using the wilcoxon rank-sum test. For the identification of marker genes over all lineages, genes associated with the Gene Ontology (GO) term for cell cycle (GO:0007049) were excluded before testing and then the top 50 ranking genes with a score > 5 used. For the characterization of endocrine subtypes and lineage formation we performed i) for each subpopulations pairwise tests against all other subpopulations (including other lineages but excluding the Lgr5+-EEC population due to the expression of many endocrine subtype markers in this cluster) and defined as specific markers all genes with a score > 5 within top 1200 ranking genes in every test, and ii) for the identification of lineage markers pairwise tests against all other subpopulations except mature endocrine subtypes and progenitor subtypes of a later stage and defined as lineage genes all genes with a score > 5 within top 1200 ranking genes in every test. With the former approach we identify genes characteristic for one subtype/stage (transient genes) while with the latter we describe genes that are specifically turned on in a lineage and then potentially sustained.

Annotation of cell types and states based on cell scores for specific signature gene sets

To obtain a cell score for a given set of signature genes the approach as described by ref[67] and implemented in the tl.score_cells function in Scanpy was used with default parameters. Shortly, the score is the average expression of the gene set subtracted with the average expression of a randomly sampled background set with expression values within the same range. For cell cycle state genes as defined in[68] were used with the tl.score_genes_cell_cycle to distinguish between G1, S and G2/M phase. For cell type scores gene sets as described in[17] were used. For metabolic signatures, genes from GO Biological processes were used (Glycolytic process: GO:0006096, Oxidative phosphorylation: GO:0042776,GO:1903862,GO:0042773). For the mitochondrial signature, genes with an mt-prefix were used. The Wnt signaling the gene set was manually curated and included *Lgr5, Ascl2, Ccnd1, Ppard, Cd44, Tcf1, Axin2, Myc, Mycn, Bmp4, Jag1, Jag2, Sox9, Ephb4, Mmp7, Fzd2, Fzd7.* For proximal and distal identity cell types were clustered with increased resolution (tl.louvain) and clusters annotated based on regional markers as described in the main text and identified in[17].

Reconstruction of lineage relationships and differentiation trajectories

To infer lineage relationships between clusters partition-based graph abstraction (PAGA) was performed using the tl.paga function of Scanpy with a threshold of 0.001 for the overall cell map and of 0.01 for the endocrine lineage[63]. Prior to PAGA the kNN-graph was recomputed on the input cell subset. PAGA generates a simple abstracted graph of clusters of the single-cell

neighborhood graph. Edge weights represent confidence of connections quantified by the connectivity of clusters. Paths in the abstracted graph rooting in the stem cell cluster represent potential differentiation trajectories. To infer directionality of differentiation a stochastic version of RNA velocity estimation as initially proposed by ref[29] was applied using the scVelo python package (ref[69], https://github.com/theislab/scvelo). Splicing information of reads (spliced/unspliced) was extracted using the velocyto pipeline (http://velocyto.org), stored in a loompy file and then read into an AnnData object for downstream analysis with scVelo and Scanpy. For the estimation of velocities and the RNA force field the recommended steps of scVelo were followed, namely: preprocessing, computing first- and second-order moments, estimating velocities, and constructing a velocity graph. First, genes were filtered, top 4000 variable genes were selected by dispersion and spliced and unspliced data layer was normalized to the initial total count per cell and log transformed. Next, first- and second-order moments were calculated for each cell across its nearest neighbors of a single-cell neighborhood-graph in PC space (number of neighbors=15, number of PCs=25). Then estimates for velocities were obtained by fitting a dynamic model of transcription for each gene. Finally, a velocity graph was computed from the correlations between potential cell transitions in the neighborhood graph and the predicted cell state change given by the velocity vector. This graph was then used to project the estimated velocities into the original low dimensional UMAP space.

Comparison of gene signatures

To calculate correlation of expression profiles between subpopulations first the average of the first 50 PCs over all cells within a subpopulation was taken and then the pairwise pearson correlation between subpopulations calculated. Clustering on correlation values was performed using the seaborn clustermap function with euclidean distance metric and average linkage method (UPGMA algorithm).

Differential expression analysis and GO term enrichment

Differential expression between conditions was performed using limma-trend[70] via an rpy2 (2.9.1) interface with batch and scaled cellular detection rate (the fraction of detected genes per cell) as additional covariates. Limma was recommended as one of the top performers in an extensive comparison and performance evaluation of available methods for differential expression methods in single-cell data[71]. Genes expressed in < 1% of cells in any of the two subsets tested were excluded. For GO term enrichment genes with a p-value < 0.01 and an estimated logFC (output from limma not the actual logFC as log transformed data was the input) > 0.1 were used. GO term enrichment was performed with the gseapy (0.9.3) implementation of EnrichR[72] and genes were weighted by their logFC value (for details see available code).

Identification of free-floating ‚ambient' mRNA

In droplet based single cell RNAseq, lysed cells in the input single cell solution can create free-floating mRNA (ambient mRNA). The ambient RNA is incorporated into the droplets with cells and is not distinguishable from endogenous mRNA after sequencing resulting in contaminating background reads mostly from highly expressed genes. For generally expressed genes this effect is expected to be similar in different samples as approximately the same total number of cells is lysed and it is not cell-type specific. However, for highly expressed marker genes of a certain cell type we observed that background expression varies between samples. This is most likely due to changed cell type frequencies, i.d. the ambient mRNA is more/less likely to come from the specific cell type. Such effects can lead to false positives in differential expression analysis between samples in cell types with only background level expression. To identify such ambient marker genes we checked for gene expression in empty droplets (1-200 total number of UMI counts) where no cell was incorporated and thus all transcripts come from free floating RNA. For each sample we considered the genes which were expressed in more than 1% of the empty droplets. We then concatenated the samples and calculated the log2 fold change of the mean of each gene between the two diet conditions. We reasoned that any gene showing a high fold change in expression between diet conditions in empty droplets would generate false positives in differential expression analyses later on. We identified *Ifitm3, H2afv, Zfos1, Smim24, Plac8, Oat, Fth1, Aldob, Defa24, Gm15284, Lyz1, Gm14851, Fabp1, Itln1, Defa17, AY761184, Gm14850, Defa20, Clps, Gm7861, Spink4, Ang4, Defa22, Defa26, Defa21, Fabp2, Gm21002, Defa23, Defa-rs1, Gm15308, Defa3, Zg16, Agr2, Tff3, Reg4, Ccl6, Defa5, Mptx2, Fcgbp, Tmsb10* as cell-type specific marker genes with a clear ambient effect (log2 fold change in empty droplets >0.15 or <-0.44). Instead of excluding these genes from differential expression tests between diet conditions we checked the distribution of the estimated logFCs (model output) for each gene over all comparisons performed. As expected for most comparisons the estimated logFCs were similar and within the ambient effect range. Outliers in these distributions were considered to be significant changes (‚above ambient effects').

Testing for changes in cell type composition

To compare the shifts in cell type proportions between two conditions a Dirichlet-Multinomial model was used. In detail, the detected cell count data was modeled with a Multinominal distribution and a Dirichlet prior on the cell type proportions as follows:

$$\mathbf{p}_c \sim \text{Dirichlet}\ (1)$$

$$\Delta_{diff} = p_{c1} - p_{c2}$$

$$\mathbf{y}_{c,i} \sim \text{Multinomial}\ (N_{c,i},\ p_c)$$

where $y_{c,i} = (y_{c,i,1}, ... y_{c,i,k})$ indicates the vector of cell counts representing the abundances of cell types in condition c, with $y_{c,i,j}$, denoting the frequency of the jth cell type, for j=1,...,k, in the ith replicate and $N_{c,i}$ being the total number of cell counts of replicate i in condition c. Δ_{diff} indicates the shift in cell type proportions between condition c_1 and condition c_2. Parameters were estimated with Hamiltonian Monte Carlo using NUTS (https://arxiv.org/abs/1111.4246) for 20,000 iterations across four chains and a burn-in of 5,000 iterations. Convergence was checked based on Gelman-Rubin convergence diagnostics[73]. Samples were drawn from the posterior distribution to compute the expected mean, standard deviation, and the 95% highest posterior density interval (HPD-region) of the frequency shift for each cell. A shift was deemed credible if the HPD-region did not include 0.

Preprocessing and analysis of scRNA-seq data of small intestinal villi

BCL files were aligned and processed using CellRanger like the scRNA-seq data of crypts described above to generate raw UMI count matrices. Barcodes with a fraction of counts from mitochondria-encoded genes greater than 25%, and genes expressed in less than 5 cells were excluded. Counts were normalized to total counts per cells using pp.normalize_total not considering highly expressed genes ('exclude_highly_expressed=True') and log-transformed. We excluded immune cells by iterative louvain-based clustering at different resolution. For each iteration a nearest-neighbor graph was recomputed on the 50 first principal components with a size of 15 neighbors. The resulting clusters of epithelial cells were annotated based on known marker genes and merged if capturing variation within the same cell type. For visualization UMAP was run.

Regionality of small intestinal villi

To group enterocytes into proximal and distal-type enterocytes, we clustered EPs and enterocytes in a low-dimensional space using a set of 44 transcription factors previously reported to be differential between proximal and distal gut regions[17]. To annotate the clusters, we computed cell scores on the set of proximal and distal transcription factors, respectively. Differential testing between CD and HFHSD enterocytes was performed using a t-test as implemented in Scanpy (tl.rank_genes_groups). We excluded a set of immune genes, which showed high background expression in all cells. Gene set enrichment was performed using the gseapy (0.10.2) implementation of EnrichR[72].

Pseudospatial ordering of cells along villus axis

To infer enterocyte zonation from villus bottom to tip, we embedded EPs and enterocytes in a low-dimensional space of 2056 zonation markers described by Moor et al[18]. We used Bbknn to compute the nearest-neighbor graph[74] to remove diet-dependent differences in the clustering. Then, we computed a pseudospatial ordering of cells from zone 1 to 5 using the diffusion pseudotime algorithm, and cell scores indicating the activation of zone-specific markers. We fitted a polynominal regression line to each of the 5 scores along the pseudospatial axis and defined the five zones based on the crossing points of the 5 lines. For instance, zone 2 starts, where the fitted "zone 2"-line crossed the "zone 1"-line (see also Extended Data Fig. 6a). Using this partitioning of the pseudospatial axis, cells were annotated to a zone.

Preprocessing of the single-cell qRT-PCR data

Processing of the single-cell qRT-PCR data was performed in R (http://www.r-project.org/). Ct values were substracted from the assumed limit of detection of the BioMark (LOD=29), which was higher than any of the Ct values which passed quality control. The expression values of the three most robustly expressed housekeeping genes *Uba52, ActB* and *Hsp90* were used as quality control measure for cells. All cells which did not express all three housekeepers were excluded. Since the three synthetic RNAs of different concentrations did not amplify within the detectable range of the BioMark system in all experimental runs, we did not use them as a reference and removed them from further analyses. All housekeeping genes (*Rn18S, ActB, Hsp90, Uba52*) and *GFP* were detected in the negative control and, therefore, were excluded from further analysis. The remaining genes were expressed in more than 3 cells. 26 missing values (due to technical problems during experimental proceedings) were then imputed with the mice function of the mice package using the Predictive Mean Matching method. Obtained cell x gene matrix of ΔCt values of 83 genes and 465 cells was input for all further analyses.

Single-cell qPCR analysis

The analysis of the single-cell qRT-PCR data was performed using Scanpy following a similar pipeline as for the scRNAseq data described above. The single-cell neighbourhood graph was calculated on the 12 first principal components with a local neighbourhood size of 25 (pp.pca and pp.neighbours). UMAP was then run for visualization (tl.umap). We used Louvain-based clustering at varying resolution for subtype annotation based on the expression of known marker genes (pp.louvain). Subtype annotation and branching trajectories of lineages and were verified in a Diffusion Map representation (not shown, the code is available upon request).

Software specifications

All analysis of single-cell RNAseq data from UMI count matrices was run with python v3.5.4 with the Scanpy v.1.0.4 and anndata v0.5.10. Versions of packages required by Scanpy that might influence numerical results were numpy v1.14.2, scipy v1.0.1, pandas v0.22.0, scikit-learn v0.19.1, statsmodels v0.8.0, python-igraph v0.7.1, louvain v0.6.1. Modelling of frequency shifts was performed using the pymc3 python package and basic tensor functionality of the theano python package. Read splicing information was extracted using loompy v2.0.12 and RNA velocity estimations were run with python v3.7.5, scVelo v.0.1.26.dev7+g5e6d395 and scanpy v.1.4.4. Villi single-cell RNAseq data was analyzed with python v3.6.12 and scanpy v.1.4.4. All figures were plotted with matplotlib and seaborn and data was exported to excel-sheets with xlsxwriter v1.0.2.
