# Supplementary material for: Diet-induced alteration of intestinal stem cell function underlies obesity and prediabetes in mice
Source: Nat Metab. 2021 Sep 22;3(9):1202–16. doi: 10.1038/s42255-021-00458-9 (PMC8458097; doi:10.1038/s42255-021-00458-9)
Supplement: Supplementary file 2 — Reporting Summary [file 42255_2021_458_MOESM2_ESM.pdf]

nature research

Corresponding author(s): Heiko Lickert, Anika Böttcher

Last updated by author(s): Aug 10, 2021

Reporting Summary

Nature Research wishes to improve the reproducibility of the work that we publish. This form provides structure for consistency and transparency in reporting. For further information on Nature Research policies, see Authors & Referees and the Editorial Policy Checklist.

Statistics

For all statistical analyses, confirm that the following items are present in the figure legend, table legend, main text, or Methods section.

n/a	Confirmed	
☐	☒	The exact sample size (*n*) for each experimental group/condition, given as a discrete number and unit of measurement
☐	☒	A statement on whether measurements were taken from distinct samples or whether the same sample was measured repeatedly
☐	☒	The statistical test(s) used AND whether they are one- or two-sided<br>*Only common tests should be described solely by name; describe more complex techniques in the Methods section.*
☐	☒	A description of all covariates tested
☐	☒	A description of any assumptions or corrections, such as tests of normality and adjustment for multiple comparisons
☐	☒	A full description of the statistical parameters including central tendency (e.g. means) or other basic estimates (e.g. regression coefficient) AND variation (e.g. standard deviation) or associated estimates of uncertainty (e.g. confidence intervals)
☐	☒	For null hypothesis testing, the test statistic (e.g. *F*, *t*, *r*) with confidence intervals, effect sizes, degrees of freedom and *P* value noted<br>*Give P values as exact values whenever suitable.*
☒	☐	For Bayesian analysis, information on the choice of priors and Markov chain Monte Carlo settings
☒	☐	For hierarchical and complex designs, identification of the appropriate level for tests and full reporting of outcomes
☐	☒	Estimates of effect sizes (e.g. Cohen's *d*, Pearson's *r*), indicating how they were calculated

Our web collection on statistics for biologists contains articles on many of the points above.

Software and code

Policy information about availability of computer code

Data collection	1. Microsoft office excel 2016-2018<br>2. qPCR was carried out using the Viia7 Real Time PCR System (Thermo Fisher Scientific).<br>3. Microarray data was obtained using Agilent 2100 Bioanalyzer and Affymetrix Mouse Gene 1.0 ST arrays.<br>4. MALDI-MSI measurements were obtained with a Bruker Solarix 7T FT-ICR-MS (Bruker Daltonics, Bremen, Germany) and after matrix removal scanned with a MIRAX DESK digital slide-scanning system (Carl Zeiss MicroImaging, Göttingen, Germany).<br>5. Western blot analysis was carried out using UVP Chem Studio SA (Analytik Jena AG, Biometra GmbH Jena Germany).<br>6. Tissue histology images were obtained with a Zeiss AXIO Scope A1 microscope (Carl Zeiss AG, Germany).<br>7. Fluorescent histological images were obtained with a Leica SP5 Confocal microscope (Leica Microsystems, Germany) using the LAS AF software v2.6.0-7266.<br>8. single-cell qRT-PCR were run on 96 × 96 Dynamic Array on the BioMark System (Fluidigm). Ct values for each gene in each cell was calculated using BioMark Real-Time PCR Analysis software v3 (Fluidigm).<br>9. single-cell libraries were sequenced on the HiSeq4000 (Illumina) with 150 bp paired-end sequencing of read 2.<br>10. BD FACS ARIA III and BD FACSDiva software v6.1.3<br>11. EchoMRI, Houston, TX, USA for body composition analysis
Data analysis	1. Prism 6 software (Graphpad) http://www.graphpad.com/scientific-software/ N/A prism/.<br>2. Microarray was processed with Expression Console (v.1.3.0.187, Affymetrix),<br>3. Spectra processing of MALDI-MSI data was done with FlexImaging v. 4.2.<br>4. MATLAB R2014b (v.7.10.0, Mathworks, Inc., Natick, MA) was used as MALDI spectral pre-processing tool for the subsequent data bioinformatics analysis, LIMPIC algorithm was used for peak picking<br>5. Metabolite annotation was performed with databases (METLIN, http://metlin.scripps.edu/; Human Metabolome Database, http://www.hmdb.ca/; MassTRIX, http://masstrix3.helmholtz-muenchen.de/masstrix3/; METASPACE,http://annotate. metaspace 2020.eu/).<br>6. Heatmap-based clustering and enrichment analysis of metabolic pathways were performed with MetaboAnalyst 3.0 (http://www.metaboanalyst.ca).<br>7. Western blots protein bands were quantified with the ImageJ software v1.51.

8. Histological images were analyzed using the LAS AF software program v2.6.0-7266..
9. scRNA-seq data were analyzed using python 3.5.4, 3.6.12 and 3.7.5. The raw reads were processed using the CellRanger analysis pipeline (v2.0.0) provided by 10X Genomics. Subsequent analyzes were carried out using custom scripts, Scanpy (https://github.com/theislab/scanpy, v1.0.4+92.g9a754bb and 1.4.4post for velocity estimation and scRNA-seq of villi cells), scVelo (0.1.26.dev7+g5e6d395, https://github.com/theislab/scvelo with scanpy v1.3.4), velocyto (v0.17.7, http://velocyto.org), limma (http://bioinf.wehi.edu.au/limma/, v3.28.10) via an rpy2 interface (v2.9.1) and gseapy (v0.9.3) implementation of EnrichR (2018/2019).
10. QIAGEN's Ingenuity Pathway Analysis (IPA®, QIAGEN Redwood City, www.qiagen.com/ingenuity).
11. CARMAweb

For manuscripts utilizing custom algorithms or software that are central to the research but not yet described in published literature, software must be made available to editors/reviewers. We strongly encourage code deposition in a community repository (e.g. GitHub). See the Nature Research guidelines for submitting code & software for further information.

Data

Policy information about availability of data

All manuscripts must include a data availability statement. This statement should provide the following information, where applicable:
- Accession codes, unique identifiers, or web links for publicly available datasets
- A list of figures that have associated raw data
- A description of any restrictions on data availability

All data generated or analysed during this study are included in this published article and its Supplementary Information files. Source data are provided with this paper.
Microarray data have been submitted to NCBI/GEO (GSE148227).
scRNAseq data have been submitted to NCBI/GEO (GSE147319).
Code and custom scripts for scRNAseq analysis have been deposited on https://github.com/theislab/2021_Aliluev_Tritschler_gut_HFD.
Databases: http://metlin.scripps.edu/; Human Metabolome Database, http://www.hmdb.ca/; MassTRIX, http://masstrix3.helmholtz-muenchen.de/masstrix3/; METASPACE,http://annotate. metaspace 2020.eu/

Field-specific reporting

Please select the one below that is the best fit for your research. If you are not sure, read the appropriate sections before making your selection.

☒ Life sciences ☐ Behavioural & social sciences ☐ Ecological, evolutionary & environmental sciences

For a reference copy of the document with all sections, see nature.com/documents/nr-reporting-summary-flat.pdf

Life sciences study design

All studies must disclose on these points even when the disclosure is negative.

Sample size

Sample size was calculated based on α-error=0.05, power=0.8, and effect size=1.58. We obtained a sample size of n=>8/condition group for physiological measurements during the high-fat high sugar diet feeding based on published data.

For single-cell RNA-seq a minimum expected requirement of ~3000 cells per experiment was used and by far exceeded in all cases for confident identification of rare cell subpopulations (~0.05% of cells). No statistical test or power analyzes were used to pre-determine sample size.
Otherwise, we chose the sample size of individual experiments based on past experience on detecting differences with a given method and relevant literature (PMID: 26935695, PMID: 22722868, PMID: 29727683).

Data exclusions

For the single-cell RNAseq data cells were filtered using previously described standards for quality control. For each experiment the count matrix was filtered as follows: for scRNA-seq samples of crypt cells genes with expression in less than 20 cells were removed and cells with a fraction of UMI counts from mitochondrially encoded genes of 10% or more were excluded, for scRNA-seq samples of villi cells genes with expression in less than 5 cells were removed and cells with a fraction of UMI counts from mitochondrially encoded genes of 25% or more were excluded. These criteria were based on the cell quality within this study. Otherwise, no data were excluded. Some mice that were on a HFHSD did not gain weight (=non-responder) and were excluded from the analysis.

Replication

Experiments were performed at least in triplicates most with three or more biological replicates unless otherwise indicated. All attempts of replication were successful. Further details about the replication of data are stated in the figure legends.

Randomization

For dietary interventions mice were randomized into test groups based on body weight distribution. For microscopy, the fields of images were randomly selected. For FACS experiments, at least 50,000 events were randomly sampled by the FACS machine. Most other results were derived from computation therefore randomization was not required.

Blinding

The investigators were not blinded to group allocation during in vivo experiments as CD and HFHSD mice were clearly distinguishable by eye. Investigators were not blinded during data collection or analysis as most experiments/analyses were performed by automatic devices such as the FACS or qPCR cycler. For cell-based assays such as Western blot or qPCR blinding was not possible because the experiments were performed by a single researcher.

Reporting for specific materials, systems and methods

We require information from authors about some types of materials, experimental systems and methods used in many studies. Here, indicate whether each material, system or method listed is relevant to your study. If you are not sure if a list item applies to your research, read the appropriate section before selecting a response.

Materials & experimental systems

n/a	Involved in the study
☐	☒ Antibodies
☒	☐ Eukaryotic cell lines
☒	☐ Palaeontology
☐	☒ Animals and other organisms
☒	☐ Human research participants
☒	☐ Clinical data

Methods

n/a	Involved in the study
☒	☐ ChIP-seq
☐	☒ Flow cytometry
☒	☐ MRI-based neuroimaging

Antibodies

Antibodies used

primary antibodies:
chicken anti-GFP (1:600, Aves Labs, USA, GFP-1020);
goat anti-ChgA (1:200, Santa Cruz, sc-1488);
rabbit anti-Lyz1 (1:1000, DAKO, M0776);
rabbit anti-Muc2 (1:1000, Santa Cruz, sc-7314);
rat anti-BrdU (1:200, Abcam, ab6326);
rabbit anti-5-HT (1:1000, Neuromics, RA20080);
anti-rabbit Ngn3 (1:100, a gift from Helena Edlund lab);
goat anti-villin (1:200, Santa Cruz, sc-7672);
goat anti-ghrelin (1:200, Santa Cruz, sc-10368);
rabbit anti-Ki67 (1:200, Abcam, ab15580);
rabbit anti-E-cadherin (extracellular domain) (1:1000, a gift from Dietmar Vestweber)
mouse anti-Srebp1 (1:1000, Novus Biologicals, NB600-582SS);
rabbit anti-Acc (1:1000, Cell Signaling Technology, 3676);
rabbit anti-Pparγ (1:1000, Cell Signaling Technology, 2435);
goat anti-lamin (1:1000, Santa Cruz, sc-6217);
mouse anti-α-tubulin (1:1000, Sigma-Aldrich ,T6199);
rabbit anti-Fasn 1:1000, Cell Signaling Technology, 3180);
rabbit anti-Scd1 (1:1000, Cell Signaling Technology, 2794);
mouse anti-β-Catenin (1:1000, BD, 610154);
rabbit anti-Gsk3β (1:5000, Cell Signaling Technology, 12456);
rabbit anti-phospho Gsk3β (1:5000, Cell Signaling Technology, 5558)

Secondary antibodies:
donkey anti-chicken Alexa Fluor 488 (1:800, Dianova, 703-225-155);
donkey anti-mouse Cy5 (1:800, Dianova, 715-175-151);
donkey anti-goat Alexa Fluor 555 (1:800, Invitrogen, A21432);
donkey anti-rabbit Alexa Fluor 555 (1:800, Invitrogen, A31572);
donkey anti-rabbit Alexa Fluor 649 (1:800, Dianova, 711-605-152);
goat anti-mouse HRP (1:15000, Dianova, 115-036-062);
goat anti-rabbit HRP (1:15000, Dianova, 111-036-045);
rabbit anti-goat HRP (1:15000, Dianova, 305-035-045)

Validation

primary antibodies: antibodies were validated by the company
chicken anti-GFP (1:600, Aves Labs, USA, GFP-1020); https://www.aveslabs.com/products/green-fluorescent-protein-gfp-antibody
goat anti-ChgA (1:200, Santa Cruz, sc-1488); https://www.scbt.com/de/p/chr-a-antibody-c-20
rabbit anti-Lyz1 (1:1000, DAKO, M0776); https://www.labome.com/product/Dako/A0099.html
rabbit anti-Muc2 (1:1000, Santa Cruz, sc-7314); https://www.scbt.com/p/mucin-2-antibody-ccp58
rat anti-BrdU (1:200, Abcam, ab6326); https://www.abcam.com/brdu-antibody-bu175-icr1-proliferation-marker-ab6326.html
rabbit anti-5-HT (1:1000, Neuromics, RA20080); https://www.neuromics.com/RA20080
anti-rabbit Ngn3 (1:100, a gift from Helena Edlund lab); as a control we used a secondary antibody only control, https://pubmed.ncbi.nlm.nih.gov/31160421/
goat anti-villin (1:200, Santa Cruz, sc-7672); https://www.scbt.com/de/p/villin-antibody-c-19
goat anti-ghrelin (1:200, Santa Cruz, sc-10368); https://www.scbt.com/p/ghrelin-antibody-c-18
rabbit anti-Ki67 (1:200, Abcam, ab15580); https://www.abcam.com/ki67-antibody-ab15580.html
rabbit anti-E-cadherin (extracellular domain) (1:1000, a gift from Dietmar Vestweber)
mouse anti-Srebp1 (1:1000, Novus Biologicals, NB600-582SS); https://www.novusbio.com/products/srebp1-antibody-2a4_nb600-582
rabbit anti-Acc (1:1000, Cell Signaling Technology, 3676); https://www.cellsignal.de/products/primary-antibodies/acetyl-coa-carboxylase-c83b10-rabbit-mab/3676

rabbit anti-Ppary (1:1000, Cell Signaling Technology, 2435); https://www.cellsignal.de/products/primary-antibodies/pparg-c26h12-rabbit-mab/2435
goat anti-lamin (Santa Cruz, sc-6217); https://www.scbt.com/de/p/lamin-b-antibody-m-20
mouse anti-α-tubulin (Sigma-Aldrich ,T6199); https://www.sigmaaldrich.com/catalog/product/sigma/t6199?lang=de&region=DE
rabbit anti-Fasn 1:1000, Cell Signaling Technology, 3180); https://www.cellsignal.de/products/primary-antibodies/fatty-acid-synthase-c20g5-rabbit-mab/3180
rabbit anti-Scd1 (1:1000, Cell Signaling Technology, 2794); https://www.cellsignal.de/products/primary-antibodies/scd1-c12h5-rabbit-mab/2794
mouse anti-β-Catenin (BD, 610154); https://www.bdbiosciences.com/us/applications/research/stem-cell-research/cancer-research/human/purified-mouse-anti--catenin-14beta-catenin/p/610154
rabbit anti-Gsk3β (1:5000, Cell Signaling Technology, 12456); https://www.cellsignal.de/products/primary-antibodies/gsk-3b-d5c5z-xp-rabbit-mab/12456
rabbit anti-phospho Gsk3β (1:5000, Cell Signaling Technology, 5558); https://www.cellsignal.de/products/primary-antibodies/phospho-gsk-3b-ser9-d85e12-xp-rabbit-mab/5558

Secondary antibodies:
donkey anti-chicken Alexa Fluor 488 (Dianova, 703-225-155); https://www.dianova.com/en/shop/703-175-155-donkey-igg-anti-chicken-igy-hl-cy5-minx-bogogphshohumsrbrtsh/
donkey anti-mouse Cy5 (Dianova, 715-175-151); https://www.dianova.com/en/shop/715-175-151-donkey-igg-anti-mouse-igg-hl-cy5-minx-bockgogphshohurbrtsh/
donkey anti-goat Alexa Fluor 555 (Invitrogen, A21432); https://www.thermofisher.com/antibody/product/Donkey-anti-Goat-IgG-H-L-Cross-Adsorbed-Secondary-Antibody-Polyclonal/A-21432
donkey anti-rabbit Alexa Fluor 555 (Invitrogen, A31572); https://www.thermofisher.com/antibody/product/Donkey-anti-Rabbit-IgG-H-L-Highly-Cross-Adsorbed-Secondary-Antibody-Polyclonal/A-31572
donkey anti-rabbit Alexa Fluor 649 (Dianova, 711-605-152); https://www.dianova.com/shop/711-605-152-esel-igg-anti-kaninchen-igg-hl-alexa-fluor-647-minx-bockgogphshohumsrtsh/
goat anti-mouse HRP (1:15000, Dianova, 115-036-062); https://www.dianova.com/shop/115-036-062-ziege-fab2-anti-maus-igg-hl-hrpo-minx-huboho/
goat anti-rabbit HRP (1:15000, Dianova, 111-036-045); https://www.dianova.com/shop/111-036-045-ziege-fab2-anti-kaninchen-igg-hl-hrpo-minx-hu/
rabbit anti-goat HRP (1:15000, Dianova, 305-035-045); https://www.dianova.com/shop/305-035-045-kaninchen-igg-anti-ziege-igg-hl-hrpo-minx-hu/

Animals and other organisms

Policy information about studies involving animals; ARRIVE guidelines recommended for reporting animal research

Laboratory animals

Homozygous Foxa2-Venus fusion (FVF) mice were generated as previously described and backcrossed to C57BL/6 background. Foxa2nEGFP-CreERT2 mice (CD1 background) were crossed with Gt(ROSA)26mTmG mice (mixed 129/SvJ, C57BL/6J background) to obtain heterozygous Foxa2nEGFP-CreERT2/+; Gt(ROSA)26 mTmG/+ animals and bred in our own facilities. Other mouse lines: Lgr5-EGFP-IRES-creERT232 (Lgr5-ki, C57BL/6J background), wild-type C57BL/6N (bred in our own facilities).
Mice were housed in groups of two to four animals and maintained at 23 ± 1 °C on a 12-hour dark/light cycle with ad libitum access to diet and water unless otherwise indicated. All experiments were performed using male animals 3 to 7 months of age.

All experiments were performed using 3-7-month-old male mice.
Mice were housed in groups of two to four animals and maintained at 23 ± 1 °C and 45-65 % humidity on a 12-hour dark/light cycle with ad libitum access to diet and water unless otherwise indicated.

Wild animals

The study did not involve wild animals

Field-collected samples

No field collected samples were used in this study.

Ethics oversight

Animal experiments were carried out in compliance with the German Animal Protection Act and with the approved guidelines of the Society of Laboratory Animals (GV-SOLAS) and of the Federation of Laboratory Animal Science Associations (FELASA). This study was approved by the institutional Animal Welfare Officer (Helmholtz Center Munich) and by the Government of Upper Bavaria, Germany.

Note that full information on the approval of the study protocol must also be provided in the manuscript.

Flow Cytometry

Plots

Confirm that:

☒ The axis labels state the marker and fluorochrome used (e.g. CD4-FITC).

☒ The axis scales are clearly visible. Include numbers along axes only for bottom left plot of group (a 'group' is an analysis of identical markers).

☒ All plots are contour plots with outliers or pseudocolor plots.

☒ A numerical value for number of cells or percentage (with statistics) is provided.

Methodology

Sample preparation	Small intestines were removed and washed with cold PBS. Villi were scraped off with a glass slide. The remaining tissue was cut into 2-cm pieces, washed several times with cold PBS and incubated in 2 mM EDTA/PBS for 35 min at 4 °C on a tube roller. Subsequently, crypts were harvested by rigorous shaking and filtered through a 70-µm mesh to remove villous contaminations/ fragments.<br>Isolated crypts were incubated with TrypLE (Life technologies, #12605) for 5 min on ice and then 5 min at 37 °C and treated with 10 µg/ml DNase in crypt complete medium (DMEM/F-12 containing 10 % FCS) for 5 min at 37 °C.<br>Single cell suspension was achieved by gentle repeated pipetting. Cells were washed twice with FACS buffer (2 % FCS, 2 mM EDTA in PBS) and pelleted at 300xg, 5 min, 4 °C. For flow cytometry, cells were collected in 1-2 ml FACS buffer supplemented with 10 µM Rock-inhibitor (Sigma-Aldrich, #Y0503) and passed through the 40 µm cell strainer caps of FACS tubes.
Instrument	Single cells were analyzed by FACS-Aria III (BD) with a 100 µm nozzle.
Software	Data were analyzed with the FACS DIVA software v6.1.3.
Cell population abundance	Abundance of intestinal cell populations (from live cells = 40-60%) are reported in this manuscript and source data (Fig. 4g, F, Extended Data Fig. 9a). When possible purity of post-sort fractions were checked by briefly re-running a small amount of sample. Purity was usually 90-95%.
Gating strategy	Single cells were gated according to their FSC-A (front scatter area) and SSC-A (side scatter area). Singlets were gated dependent on the FSC-W (front scatter width) and FSC-H (front scatter height) and SSC-W and SSC-H and dead cells were excluded using the marker7AAD (eBioscience). To obtain FVF-enriched small intestinal crypt cell samples for single-cell RNA sequencing, 30,000 FVF + (FVFlow and FVFhi) cells were sorted followed by sorting 30,000 live crypt cells per sample.

☒ Tick this box to confirm that a figure exemplifying the gating strategy is provided in the Supplementary Information.
