# Supplementary figures and images for: Diet-induced alteration of intestinal stem cell function underlies obesity and prediabetes in mice
Source: Nat Metab. 2021 Sep 22;3(9):1202–16. doi: 10.1038/s42255-021-00458-9 (PMC8458097; doi:10.1038/s42255-021-00458-9)

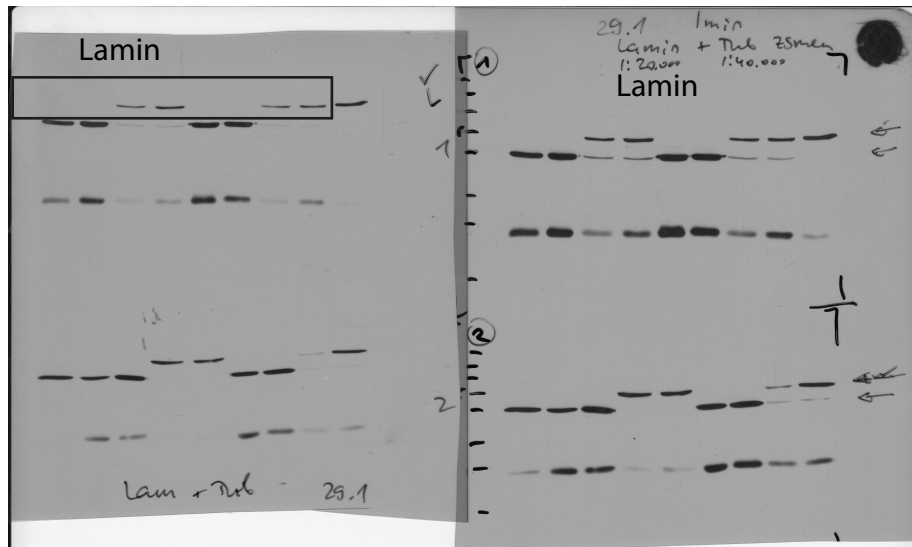

Ppary

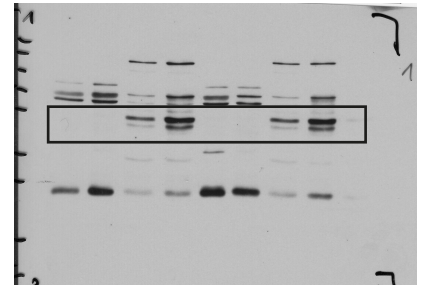

Sreb

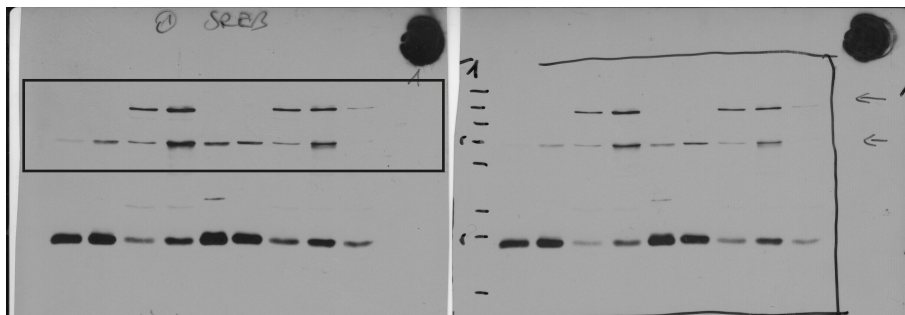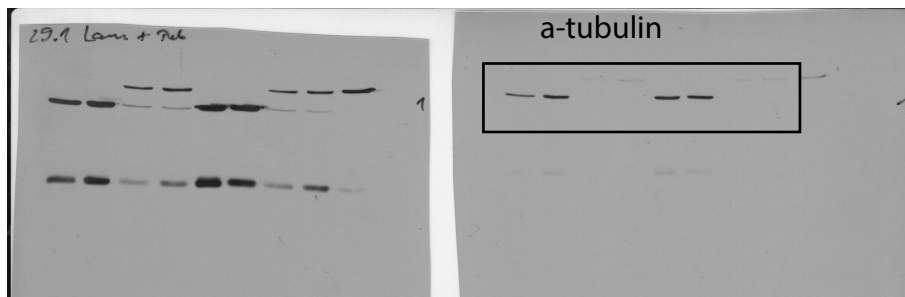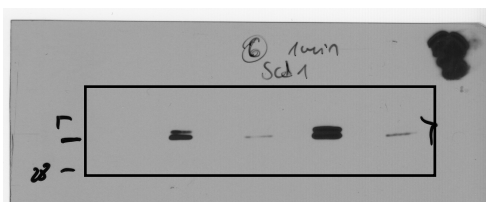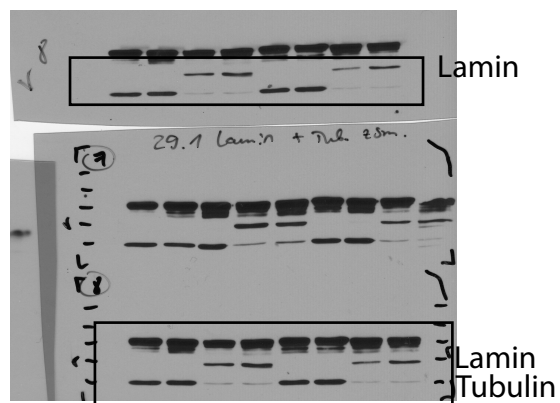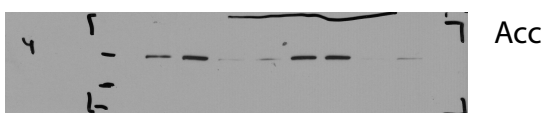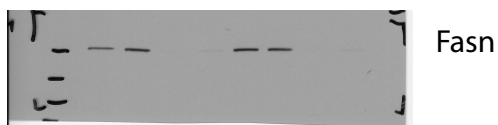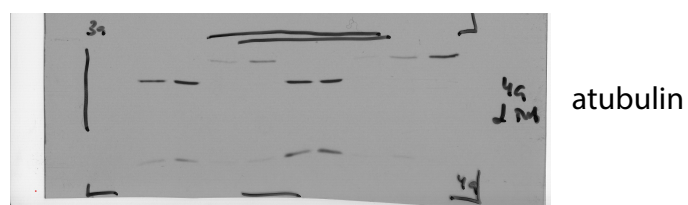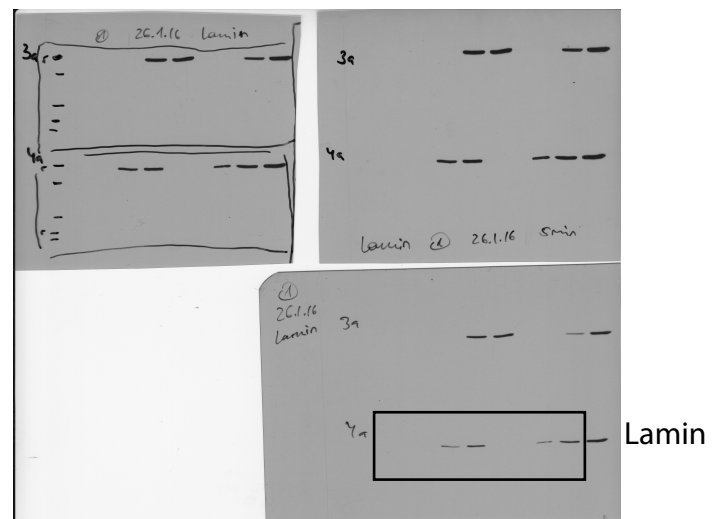

Supplement: Source Data Fig. 5 — Unprocessed western blot. [file 42255_2021_458_MOESM18_ESM.pdf]

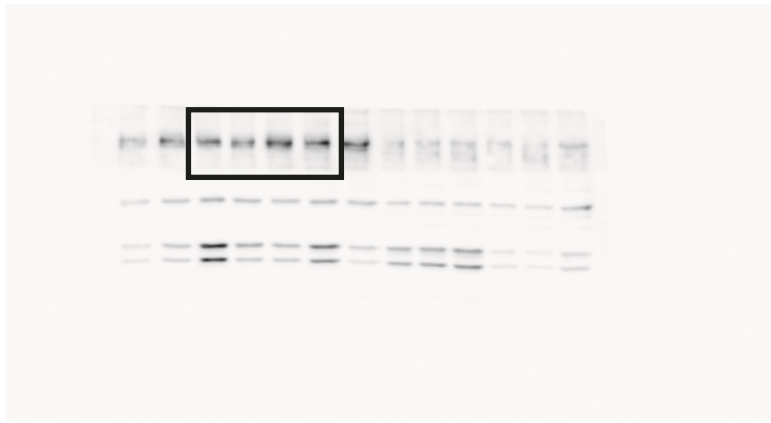

plgf1r/pInsr

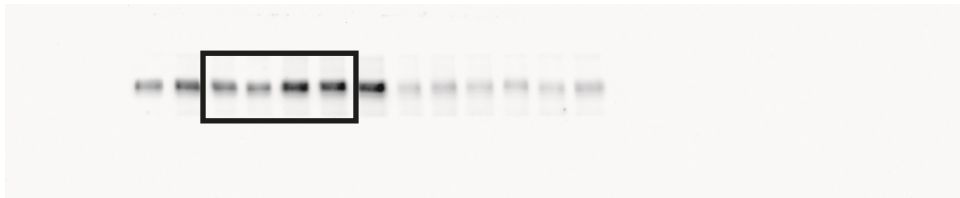

Igf1r

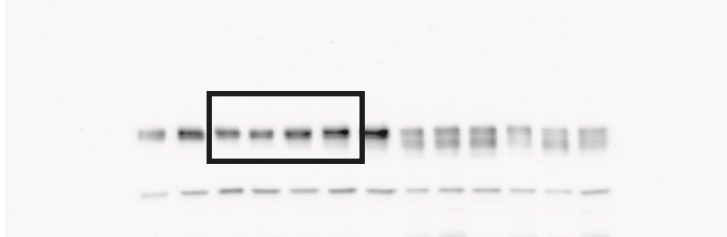

Insr-beta

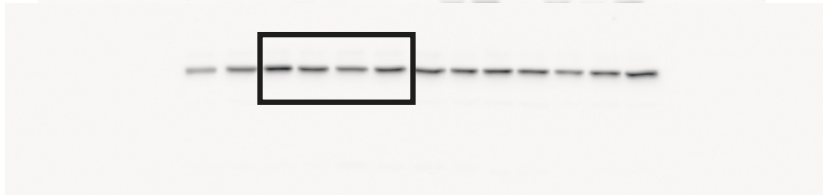

tubulin

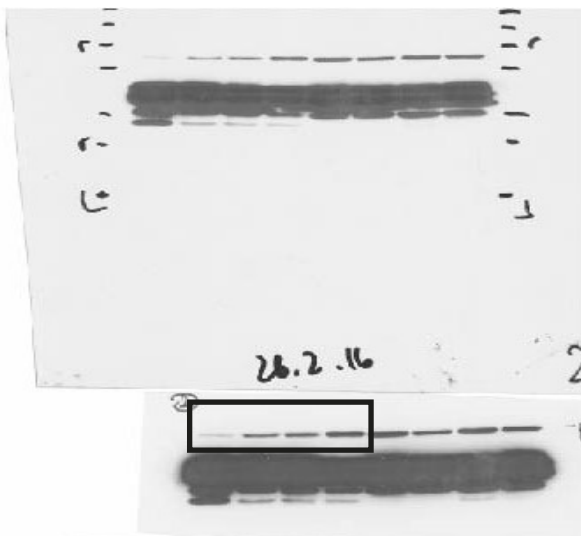

pAkt

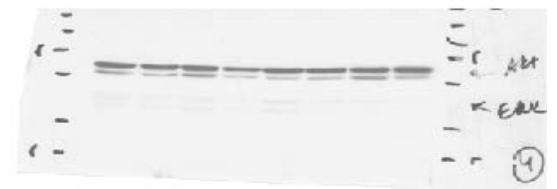

Akt

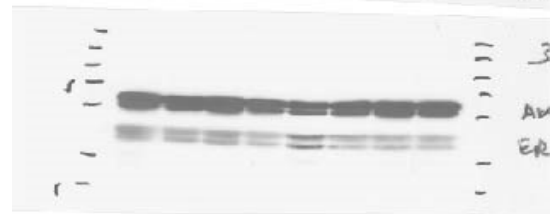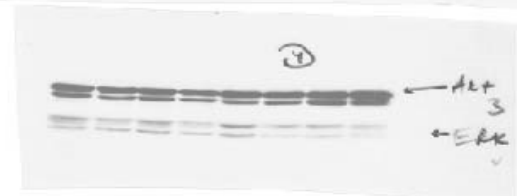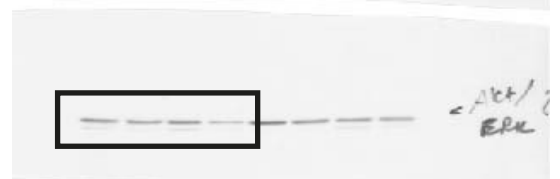

Akt

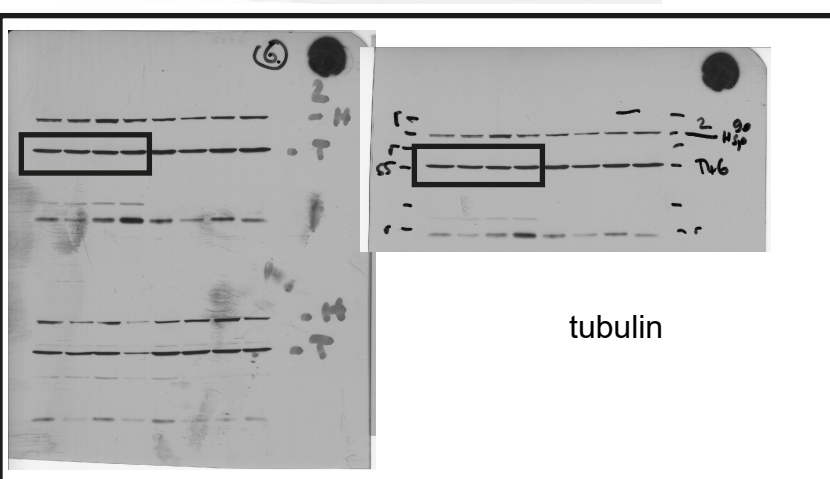

tubulin

Supplement: Source Data Extended Data Fig. 9 — Statistical source data for unprocessed western blot. [file 42255_2021_458_MOESM25_ESM.pdf]
